# Stochastic Processes Drive the Assembly and Metabolite Profiles of Keystone Taxa during Chinese Strong-Flavor Baijiu Fermentation

**DOI:** 10.1128/spectrum.05103-22

**Published:** 2023-03-14

**Authors:** Shukun Yuan, Hai Du, Dong Zhao, Zongwei Qiao, Jia Zheng, Xiaowei Yu, Yan Xu

**Affiliations:** a Laboratory of Brewing Microbiology and Applied Enzymology, Key Laboratory of Industrial Biotechnology of the Ministry of Education, School of Biotechnology, Jiangnan University, Wuxi, Jiangsu, China; b Wuliangye Yibin Co., Ltd., Yibin, Sichuan, China; University of Minnesota—Twin Cities

**Keywords:** keystone taxa, community assembly, stochastic processes, deterministic processes, metabolic profiles, microbial network

## Abstract

Multispecies communities participate in the fermentation of Chinese strong-flavor Baijiu (CSFB), and the metabolic activity of the dominant and keystone taxa is key to the flavor quality of the final product. However, their roles in metabolic function and assembly processes are still not fully understood. Here, we identified the variations in the metabolic profiles of dominant and keystone taxa and characterized their community assembly using 16S rRNA and internal transcribed spacer (ITS) gene amplicon and metatranscriptome sequencing. We demonstrate that CSFB fermentations with distinct metabolic profiles display distinct microbial community compositions and microbial network complexities and stabilities. We then identified the dominant taxa (Limosilactobacillus fermentum, Kazachstania africana, Saccharomyces cerevisiae, and Pichia kudriavzevii) and the keystone ecological cluster (module 0, affiliated mainly with Thermoascus aurantiacus, Weissella confusa, and Aspergillus amstelodami) that cause changes in metabolic profiles. Moreover, we highlight that the alpha diversity of keystone taxa contributes to changes in metabolic profiles, whereas dominant taxa exert their influence on metabolic profiles by virtue of their relative abundance. Additionally, our results based on the normalized stochasticity ratio (NST) index and the neutral model revealed that stochastic and deterministic processes together shaped CSFB microbial community assemblies. Stochasticity and environmental selection structure the keystone and dominant taxa differently. This study provides new insights into understanding the relationships between microbial communities and their metabolic functions.

**IMPORTANCE** From an ecological perspective, keystone taxa in microbial networks with high connectivity have crucial roles in community assembly and function. We used CSFB fermentation as a model system to study the ecological functions of dominant and keystone taxa at the metabolic level. We show that both dominant taxa (e.g., those taxa that have the highest relative abundances) and keystone taxa (e.g., those taxa with the most cooccurrences) affected the resulting flavor profiles. Moreover, our findings established that stochastic processes were dominant in shaping the communities of keystone taxa during CSFB fermentation. This result is striking as it suggests that although the controlled conditions in the fermentor can determine the dominant taxa, the uncontrolled rare keystone taxa in the microbial community can alter the resulting flavor profiles. This important insight is vital for the development of potential manipulation strategies to improve the quality of CSFB through the regulation of keystone species.

## INTRODUCTION

Microbes are closely related to the performance of many ecosystems, including human and plant health (1, 2), agricultural production (3, 4), and fermented food quality (5, 6). Since these ecosystems are hosts to a vast diversity of microbes, it is challenging to link patterns of microbial diversity with the ecological processes that generate these patterns as well as ecosystem function (7–9). Recent studies have noted the existence of dominant taxa (taxa with high relative abundances) and keystone taxa (taxa with high connectivity in the microbial network) in complex microbial communities (10). Keystone taxa may differ from dominant taxa in their levels of connectivity within and across communities and exert a more obvious influence on microbiome function than dominant taxa (10–12). Previous opinion articles stated that keystone taxa might exert their influence on microbiome function irrespective of abundance (10). Yet empirical evidence to support this opinion is lacking, and the mechanisms by which keystone taxa influence microbiome function remain largely unexplored. In addition, evidence is mounting that both deterministic processes such as abiotic and biotic selection (5, 13) as well as stochastic processes such as dispersal and ecological drift (14–17) govern the assembly of microbial communities. However, the relative contributions of deterministic and stochastic processes to the assemblies of keystone taxa remain largely unexplored. Improving our understanding of the assembly processes of keystone taxa and the mechanisms by which keystone taxa influence microbiome function is of key importance for understanding the relationship between microbial diversity and microbiome function.

In order to assess the assembly process that shapes the distribution of keystone taxa and how it might affect microbiome function, we focused on the Chinese strong-flavor Baijiu (CSFB) fermentation system, a spontaneous solid-state fermentation (SSF) process (18–21). Microorganisms from various sources (e.g., the environment, tools, the ground, and Daqu) can colonize fermented grains (FGs) (Jiupei in Chinese) to form a multispecies microbial community consisting of bacteria (e.g., *Bacillus* and *Lactobacillus*), yeasts (e.g., *Saccharomyces*, *Pichia*, and *Candida*), and molds (e.g., Aspergillus, *Mucor*, and *Rhizopus*) (21–23). This diverse microbiota primarily drives the biochemical reaction processes of starch saccharification, alcohol fermentation, and flavor compound synthesis, resulting in the conversion of raw materials into flavor substrates such as esters, alcohols, fatty acids, aldehydes, and ketones (21, 24). In addition, these communities are reproducibly cultivated on a known substrate and form multiple repeating units, allowing sampling and measurement *in situ* as well as isolation and culture *in vitro* (25). Thus, this reproducible, accessible, and culturable system (18) might be a valuable model for investigating the mechanisms of microbial community formation and the relationship between microbial community composition and function.

The fermentation system for CSFB is a typical seminatural microbial ecosystem distinguished by its openness and spontaneity. During fermentation, changeable environmental factors, including temperature (20°C to 35°C), moisture (55 to 75%), and acidity (1.5 to 3.5 nmol/10 g), as well as temporal and spatial scales provide a wide variety of ecological niches for diverse microbial communities (19). Previous works provided a detailed view of the diversity and metabolic function of dominant taxa during CSFB fermentation by culture-dependent and culture-independent approaches (21, 26–28). Among these taxa, *Lactobacillus* is the dominant lactic acid bacterium in FGs, accounting for >50% of the relative abundance of the total bacterial communities, and contributes directly to lactic acid and ethyl lactate production (29). Both *Saccharomyces* and *Pichia* are the main yeasts isolated from fermented grains and contribute to the formation of ethanol and flavor compounds (such as ethyl acetate [EA], isobutyl alcohol, isoamyl alcohol, and 2-phenethyl alcohol), respectively (27). Aspergillus and *Rhizopus* are the dominant molds in FGs and act as both saccharifying agents and flavor compound producers, attributed to their ability to secrete glucoamylase, α-amylase, proteases, and lipase (19). Moreover, several studies demonstrated that the dispersal and colonization of environmental microbiota (22), abiotic factors (e.g., pH, temperature, anaerobic environment, and moisture) (30, 31), and biotic interactions (32, 33) all contribute to the formation of dominant taxa. For instance, air-sourced Lactobacillus acetotolerans can survive under abiotic selection in a strongly acidic environment and eventually becomes the dominant species (34). Volatile compound-mediated microbial interactions between Pichia kudriavzevii and Monascus purpureus were beneficial for maintaining the diversity of yeast and ethanol metabolism (35). Yet keystone taxa are often ignored. It remains unknown if variations in keystone taxa in complex microbial communities generate quality fluctuations apparent in the metabolic profile during CSFB fermentation. Furthermore, the ecological process shaping the distribution of keystone taxa during CSFB fermentation is much less clear. More specifically, little is known about the relative importance of deterministic and stochastic processes in shaping the distribution and structure of the keystone taxa during CSFB fermentation.

Here, we collected fermented grain samples during CSFB fermentation in different environments. First, we examined their metabolic profiles, microbial community structures, and microbial networks to identify the dominant and keystone taxa responsible for metabolite variations. Next, SSF of CSFB was performed on a synthetic community incorporating dominant and keystone species isolated from FGs to confirm the importance of keystone species in the formation of metabolic profiles. Finally, we used multiple statistical approaches to disentangle the assembly rules for dominant and keystone taxa.

## RESULTS

### Comparison of metabolic profiles in fermented grains from different workshops.

**FIG 1:**
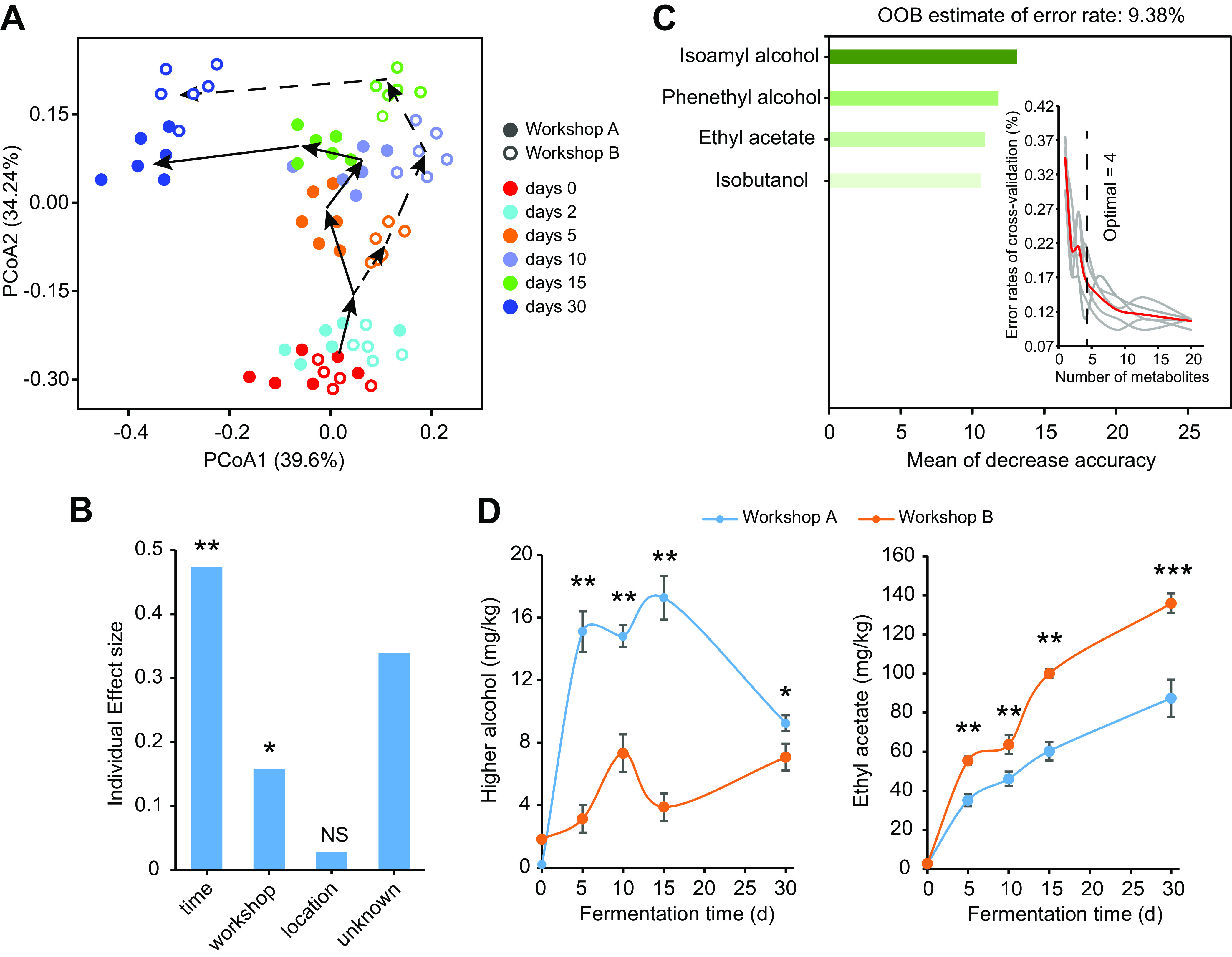
Metabolic profiles during CSFB fermentation. (A) Principal-coordinate analysis (PCoA) visualizing metabolite variations based on Bray-Curtis distance between workshops A and B (*n* = 36 fermented grain samples for each workshop). (B) Individual effect sizes of fermentation times, different workshops, and sampling locations on the metabolic profiles based on PERMANOVA. (C) Random-forest model identifying indicator metabolites that distinguish the metabolic profiles of workshops A and B. OOB (out of the bag) is an estimate of the error rate. The inset represents the 10-fold cross-validation error as a function of the number of input metabolites used to differentiate workshop A and B metabolic profiles in order of variable importance. (D) Changes in indicator metabolite contents during fermentation. Higher alcohols are the general term for the compounds isoamyl alcohol, phenethyl alcohol, and isobutanol. Data are presented as the means ± standard deviations. *, *P < *0.05; **, *P < *0.01; ***, *P < *0.001; NS, not significant (by Tukey’s test).

We used fermented grain samples that harbor distinct metabolic profiles to study the dominant and keystone taxa responsible for metabolic fluctuations. To ensure the distinctness of their metabolic profiles, samples were obtained from six batches of fermentation in two workshops (as described in Materials and Methods). A total of 72 fermented grain samples (36 fermented grain samples from each workshop) during the fermentation process were collected. We monitored metabolite concentrations in all samples (see Fig. S1A in the supplemental material). Principal-coordinate analysis (PCoA) showed that the metabolic profiles of fermented grains from both workshops A and B clustered according to fermentation times (Fig. 1A). Furthermore, the metabolic profiles of fermented grains from workshops A and B showed distinct successional trajectories after fermentation for 5 days (Fig. 1A). Although the fermentation time explained 47.4% of the variance in metabolic profiles across these samples (*R*^2^ = 0.474; *P < *0.001 [by permutational multivariate analysis of variance {PERMANOVA}]) (Fig. 1B), different workshops also contributed 15.7% of the total variation (*R*^2^ = 0.157; *P < *0.001 [by PERMANOVA]) (Fig. 1B). As evidenced by PERMANOVA, the metabolic profiles of workshops A and B were significantly different at days 5, 10, and 15 (see Fig. S7 at https://doi.org/10.6084/m9.figshare.21379119). Next, a random-forest machine learning method was used to identify biomarkers that distinguish the metabolic profiles of workshops A and B. Our model showed a 90.62% accuracy of the metabolic profile classifications. We carried out 10-fold cross-validation to evaluate the importance of indicator metabolites in fermented grains. The cross-validation error curve stabilized when the 4 most relevant metabolites were included (Fig. 1C). Thus, we defined these 4 metabolites (isoamyl alcohol, phenethyl alcohol, ethyl acetate [EA], and isobutanol) as indicator metabolites. Of these, 3 higher alcohols (HAs) (isoamyl alcohol, phenethyl alcohol, and isobutanol) showed higher concentrations in fermented grains from workshop A than in those form workshop B after 5 days of fermentation; EA showed higher concentrations in fermented grains from workshop B than in those from workshop A (Fig. 1D; see also Table S2 at https://doi.org/10.6084/m9.figshare.21382410). Taken together, our results indicated that fermented grain samples from different workshops vary in their metabolic profiles and that the variation was explained mainly by EA and HA variations.

### Targeting the potential dominant members of the microbiota responsible for changes in metabolic profiles.

**FIG 2:**
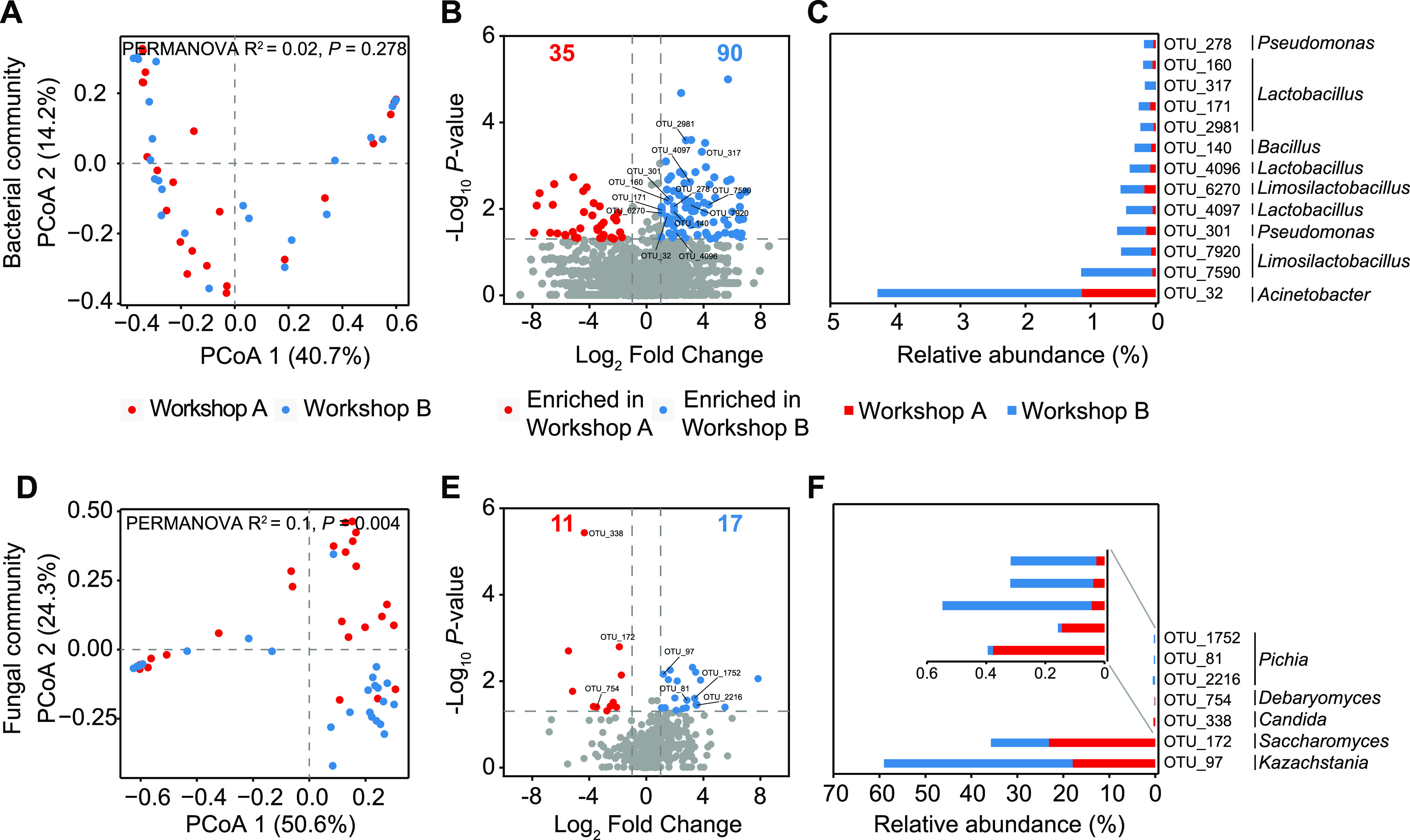
Differences in microbial communities between workshops. (A and D) PCoA plots were used to visualize the differences in bacterial and fungal community compositions based on the Bray-Curtis distance between workshops A and B (*n* = 24 fermented grain samples for each workshop). (B and E) Volcano plots displaying members of the microbiota enriched in either workshop A or B. (C and F) Enriched OTUs with a relative abundance of >0.1%.

To identify the members of the microbiota that have the potential to cause variations in metabolic profiles, we analyzed the differences in the overall microbial community compositions of 48 fermented grain samples by 16S rRNA and internal transcribed spacer (ITS) gene amplicon sequencing (Fig. S1A). Overall, PCoA and PERMANOVA showed that the bacterial community compositions in the two workshops did not differ significantly (*R*^2^ = 0.02; *P *= 0.278) (Fig. 2A), while the fungal community compositions exhibited a clear separation (*R*^2^ = 0.1; *P *= 0.004) (Fig. 2D). In general, the bacterial and fungal communities in both workshops clustered according to fermentation times (Fig. S2B and D). However, we found that the fungal communities in both workshops also exhibited distinct successional trajectories (Fig. 2D). This result was consistent with the variation in the metabolic profiles. We then observed the temporal patterns of the microbial community structures during fermentation (Fig. S2A and C). For bacteria, *Weissella*, *Pelomonas*, *Limosilactobacillus*, and *Lactobacillus* were the dominant bacteria on day 0. During the fermentation process, the abundances of *Weissella* and *Pelomonas* decreased, while the abundances of *Lactobacillus*, *Limosilactobacillus*, and *Secundilactobacillus* increased gradually. The relative abundance of *Acetilactobacillus* increased dramatically and occupied 80% after 30 days of fermentation (Fig. S2A). For fungi, *Thermoascus*, Aspergillus, and *Thermomyces* dominated the fungal community on day 0; however, their abundances dramatically decreased after 2 days of fermentation. *Kazachstania* and *Saccharomyces* were the dominant fungal genera throughout fermentation. The relative abundance of *Kazachstania* dramatically increased from 1.7% on day 0 to 66% on day 5 and then gradually decreased to 60.9% from day 10 to day 30. *Candida* was more dominant in fermented grain samples from workshop A after 2 days of fermentation, while *Kazachstania* was more abundant in those from workshop B (Fig. S2C).

We used a volcano plot analysis to identify operational taxonomic units (OTUs) that were specifically enriched in each workshop. For the bacterial community, 35 and 90 bacterial OTUs were enriched in workshops A and B, respectively (Fig. 2B). Among them, dominant bacterial OTUs (relative abundance of >0.1%) were targeted, which belonged to 5 genera (Acinetobacter, *Lactobacillus*, *Limosilactobacillus*, Pseudomonas, and *Bacillus*). These 5 genera were more abundant in workshop B (Fig. 2C). For the fungal community, 11 and 17 fungal OTUs were enriched in workshops A and B, respectively (Fig. 2E). Among them, dominant fungal OTUs (relative abundance of >0.1%) were targeted, which belonged to 5 genera (*Kazachstania*, *Saccharomyces*, *Candida*, *Debaryomyces*, and *Pichia*). *Kazachstania* and *Pichia* were more abundant in workshop B, while the other three genera were more abundant in workshop A (Fig. 2F). These alterations in microbial abundance may be responsible for the different metabolic profiles observed.

### Metatranscriptome analysis confirmed the dominant members of the microbiota leading to variation in metabolic profiles.

**FIG 3:**
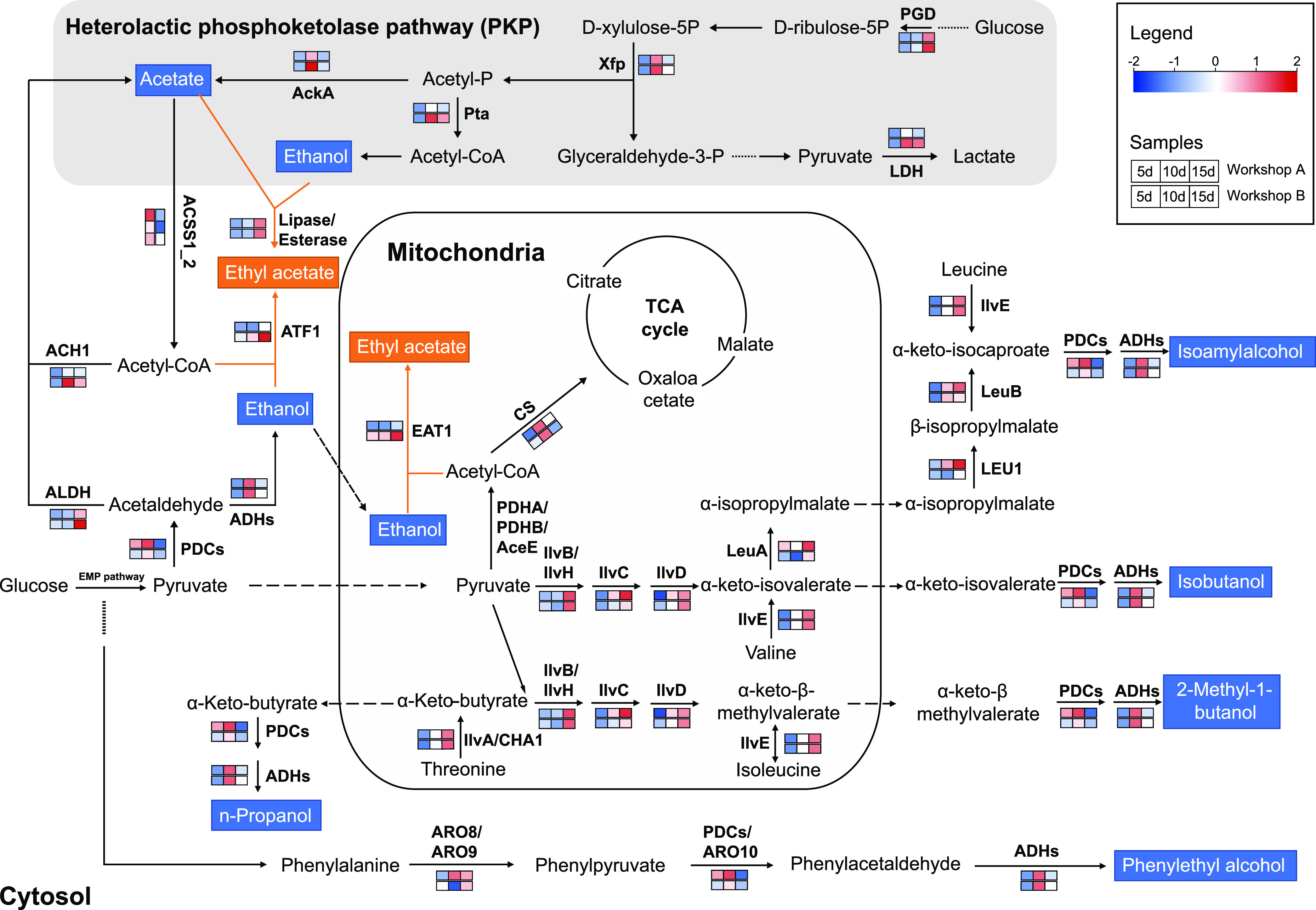
Metabolic pathway analysis of the production of ethyl acetate and higher alcohols on days 5, 10, and 15 of CSFB fermentation. Relative expression levels (transcripts per million [TPM]) of key enzyme-related genes are shown (red, high expression levels; blue, low expression levels). The enzymes involved are pyruvate decarboxylase (PDC) (K01568), aldehyde dehydrogenase (ALDH) (K00128), acetyl-CoA synthetase (ACSS1_2) (K01895), alcohol dehydrogenases (ADHs) (K00001 and K00002, etc.), alcohol *O*-acetyltransferase 1 (ATF1) (K00664), ethanol acetyltransferase 1 (EAT1) (K22586), citrate synthase (CS) (K01647), acetolactate synthase I/II/III large subunit (IlvB) (K01652), acetolactate synthase I/III small subunit (IlvH) (K01653), ketol acid reductoisomerase (IlvC) (K00053), dihydroxy acid dehydratase (IlvD) (K01687), 2-isopropylmalate synthase (LeuA) (K01649), 3-isopropylmalate dehydratase (LEU1) (K01702), 3-isopropylmalate dehydrogenase (LeuB) (K00052), branched-chain amino acid aminotransferase (IlvE) (K00826), threonine deaminase (IlvA) (K01754), l-serine/l-threonine ammonia-lyase (CHA1) (K17989), aromatic amino acid aminotransferase I (ARO8) (K00838), aromatic amino acid aminotransferase II (ARO9) (K05821), phenylpyruvate decarboxylase (ARO10) (K12732), pyruvate dehydrogenase E1 component alpha subunit (PDHA) (K00161), pyruvate dehydrogenase E1 component beta subunit (PDHB) (K00162), pyruvate dehydrogenase E1 component (AceE) (K00163), 6-phosphogluconate dehydrogenase (PGD) (K00033), xylulose-5-phosphate (Xfp) (K01621), acetate kinase (AckA) (K00925), phosphate acetyltransferase (Pta) (K00625), l-lactate dehydrogenase (LDH) (K00016), and acetyl-CoA hydrolase (ACH1) (K01067). EMP pathway, Embden-Meyerhof-Parnas pathway; TCA, tricarboxylic acid. The number in brackets is the accession number of enzyme in the KEGG database (https://www.genome.jp/kegg/).

To further determine the dominant members of the microbiota and functional genes that cause changes in the indicator metabolites, we analyzed the microbial metabolic functions in 10 fermented grain samples by metatranscriptomics (Fig. S1A). A metabolic network of EA and HAs during fermentation was constructed using the annotated enzymes and their metabolic pathways (Fig. 3; see also Table S8 at https://doi.org/10.6084/m9.figshare.21382410). The key enzymes related to ethyl acetate biosynthesis were alcohol *O*-acetyltransferase 1 (ATF1) and ethanol acetyltransferase 1 (EAT1) (36, 37). In this study, the genes for ATF1 (K00664) and EAT1 (K22586) showed the highest levels of expression in Kazachstania africana, followed by Saccharomyces cerevisiae and *Pichia kudriavzevii* (Fig. S3A). Furthermore, genes related to esterase (*est*) (K03928) were also annotated. The highly expressed members of these enzymes originated mostly from Aspergillus ruber, Limosilactobacillus fermentum, and K. africana (Fig. S3B). As one of the important precursors of ethyl acetate synthesis, acetic acid can be synthesized by the heterolactic phosphoketolase pathway (PKP) in heterofermentative lactobacilli (38). The key enzymes related to the PKP included 6-phosphogluconate dehydrogenase (PGD) (K00033), xylulose-5-phosphate (Xfp) (K01621), and acetate kinase (AckA) (K00925). Highly expressed members of these enzymes originated mainly from L. fermentum, Levilactobacillus koreensis, Apilactobacillus kunkeei, and Limosilactobacillus oris (Fig. S3F).

There were two metabolic pathways involved in the biosynthesis of higher alcohols, including the Ehrlich pathway and the anabolic pathway (39). The key enzymes related to the Ehrlich pathway included branched-chain amino acid aminotransferase (IlvE) (K00826), threonine deaminase (IlvA) (K01754), l-serine/l-threonine ammonia-lyase (CHA1) (K17989), and aromatic amino acid aminotransferase I/II (ARO8) (K00838), which catalyze the decomposition of amino acids to produce α-keto acid (Fig. 3). We found that the highly expressed members of these enzymes originated mostly from Nakaseomyces bacillisporus, *K. africana*, S. cerevisiae, and *L. fermentum* (Fig. S3C). Previous studies have confirmed that most enzymes related to the anabolic pathway are shared with the branched-chain amino acid synthesis pathway (isoleucine-leucine-valine [ILV] biosynthesis pathway) (40). Among them, the acetolactate synthase I/II/III large subunit (IlvB) (K01652), ketol acid reductoisomerase (IlvC) (K00053), and dihydroxy acid dehydratase (IlvD) (K01687) were key enzymes for the synthesis of isobutanol and 2-methyl-1-butanol (Fig. 3). Highly expressed members of these enzymes originated from Naumovozyma castellii, Lachancea quebecensis, S. cerevisiae, *K. africana*, and *P. kudriavzevii* (Fig. S3D). In addition, 2-isopropylmalate synthase (LeuA) (K01649), 3-isopropylmalate dehydratase (LEU1) (K01702), and 3-isopropylmalate dehydrogenase (LeuB) (K00052) were three key enzymes involved in isoamyl alcohol production (Fig. 3). Highly expressed members of these enzymes originated from *K. africana*, S. cerevisiae, *P. kudriavzevii*, and *L. fermentum* (Fig. S3E).

The dominant members of the microbiota responsible for metabolic changes were determined based on the following criteria: (i) significant changes in abundance across different workshops as determined by amplicon sequencing and (ii) the ability to express key enzymes related to EA and HA synthesis at the transcriptional level. Accordingly, 3 bacterial OTUs and 5 fungal OTUs were targeted, which belonged to 4 species (*L. fermentum*, *K. africana*, S. cerevisiae, and *P. kudriavzevii*) (Fig. S3G).

### Microbial network complexity and stability.

**FIG 4:**
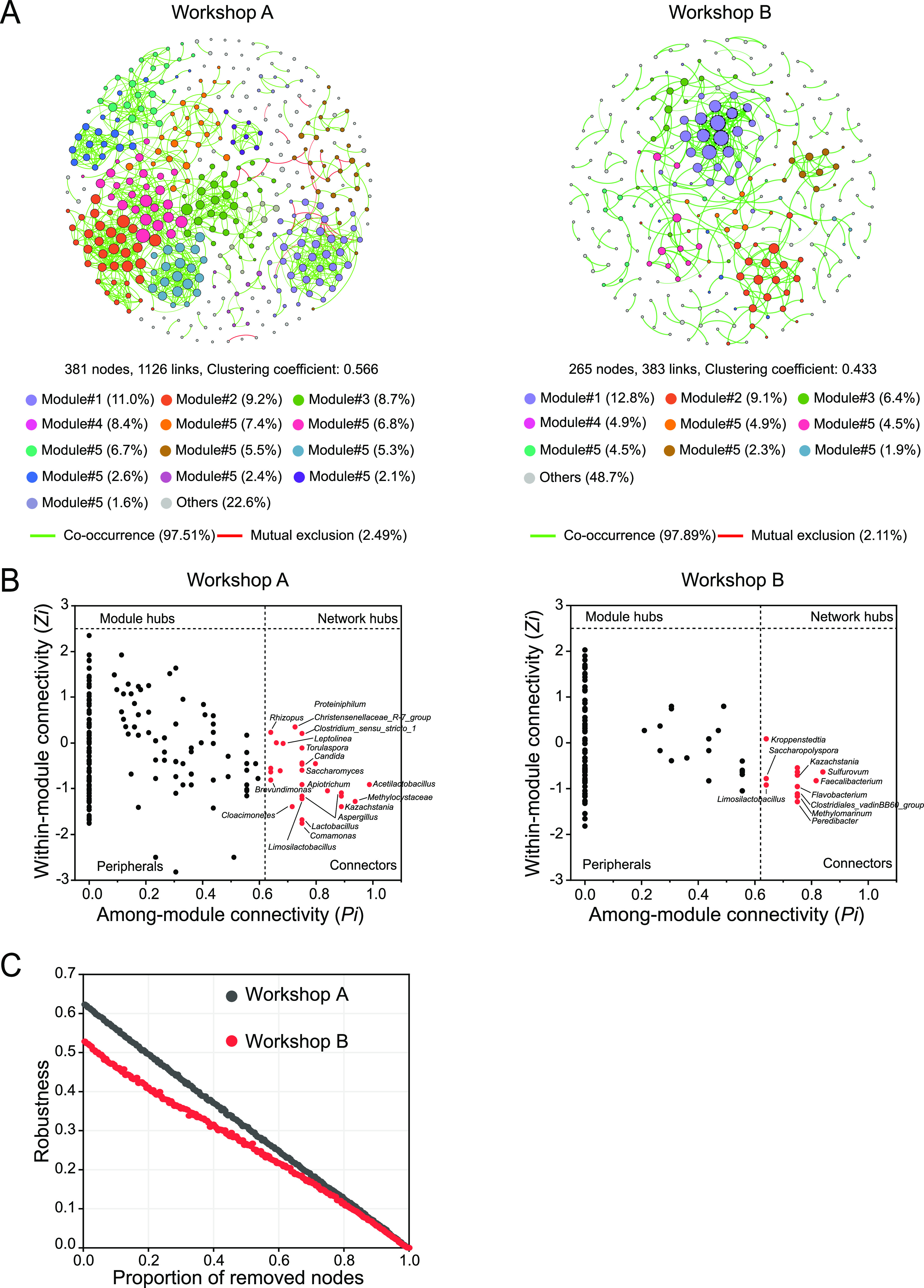
Microbial network complexity and stability. (A) Networks of cooccurrence patterns between bacterial and fungal taxa at the OTU level for workshops A and B (*n* = 24 fermented grain samples for each workshop). The node size is proportional to the degree of connection, and the nodes are colored according to modules. Only modules with at least 5 nodes are shown. The edges are colored according to interaction types; positive correlations are labeled in green, and negative correlations are shown in red. (B) Putative keystone taxa identified based on the topological roles of the nodes in networks in workshops A and B. Each symbol represents a node in one of the networks. A node was identified as a module hub if its *Z_i_* was ≥2.5, as a connector if its *P_i_* was ≥0.62, and as a network hub if it had a *Z_i_* of ≥2.5 and a *P_i_* of ≥0.62. (C) Robustness analysis showing the relationships between the proportion of the remaining species in the network and the proportion of the taxa randomly removed in microbial networks. The same proportion of node removal results in a greater decrease in robustness, which means lower network stability.

In order to identify differences in microbiome associations, two networks were constructed for the two workshops (workshop A and workshop B) by combining all microbiomes originating from the samples (*n* = 48) (Fig. 4A). Although the numbers of OTUs used for network construction were same for both workshops, the numbers of network nodes and links in workshop A were 44% and 194% larger than those in workshop B (Table S1), respectively, suggesting that these microbial taxa could be associated more closely with each other in workshop A. The clustering coefficient, density, and modularity of the network in workshop A were increased by 0.133, 0.005, and 0.028, respectively, in comparison with those in workshop B (Table S1). Network module detection was carried out, and different module distributions were found between the two workshops. A total of 13 large modules (modules with ≥5 nodes) (41) accounted for 77.4% of the nodes in the network in workshop A, while 9 large modules accounted for 51.3% of the nodes in the network in workshop B (Fig. 4A; Table S1), suggesting that more nodes in workshop A tend to form highly connected local structures than in workshop B. Altogether, these results indicated that the microbial network in workshop A consistently had more complexity than the microbial network in workshop B.

The identification of potential keystone nodes wase performed according to criteria reported previously, which defined network hubs, module hubs, connectors, and peripherals based on their within-module connectivity (*Z_i_*) and among-module connectivity (*P_i_*) (41). Totals of 27 and 12 connectors (nodes linking different modules) were detected in the networks in workshop A and workshop B, respectively (Fig. 4B), all of which could be regarded as keystone nodes. Importantly, there were 56% more keystone nodes in workshop A than in workshop B, and the proportion of keystone fungal nodes in workshop A (37%) was higher than that in workshop B (0.8%). Therefore, we can confirm that workshop A and workshop B have different microbial network structures at the keystone node level. In addition, we examined the distributions of the relative abundances of keystone nodes. Their relative abundances spanned a range from low to high (0.0054 to 3.38%), but most of them were not among the most abundant taxa in the communities (see Fig. S7 at https://doi.org/10.6084/m9.figshare.21379119).

We simulated species extinction and calculated the robustness (resistance to node loss) of the networks (41). We found that the remaining species in the microbial network decreased to a greater degree, with a greater fluctuation in workshop B than in workshop A, by removing the same proportion of nodes, showing consistently higher robustness in workshop A than in workshop B (Fig. 4C). Collectively, the above-described results indicated that the stability of the microbial network in workshop A was relatively high, probably due to the higher network complexity associated with connectivity, modularity, and keystone taxa.

### Keystone taxa in ecological clusters linked to CSFB metabolites.

Now that we have discovered the significantly different microbial community network complexities viewed at the level of keystone taxa and metabolic profiles between workshops A and B, an intriguing question is, Which keystone taxa account for the most variations in the metabolites? An ecological network provides critical information on the potential associations among thousands of organisms and can be a powerful tool for inferring keystone taxa associated with microbial community function (10). We thus constructed an ecological network by combining all microbiomes originating from the two workshops (*n* = 48) to identify clusters of microbial taxa linked to metabolites by linear regression. Since the diversity of keystone taxa within specific ecological clusters, rather than the relative abundance, has recently been highlighted as a major driver of microbiome function (3, 10), we use alpha diversity (Shannon diversity index [SDI]) to establish relationships with metabolite concentrations during CSFB fermentation.

**FIG 5:**
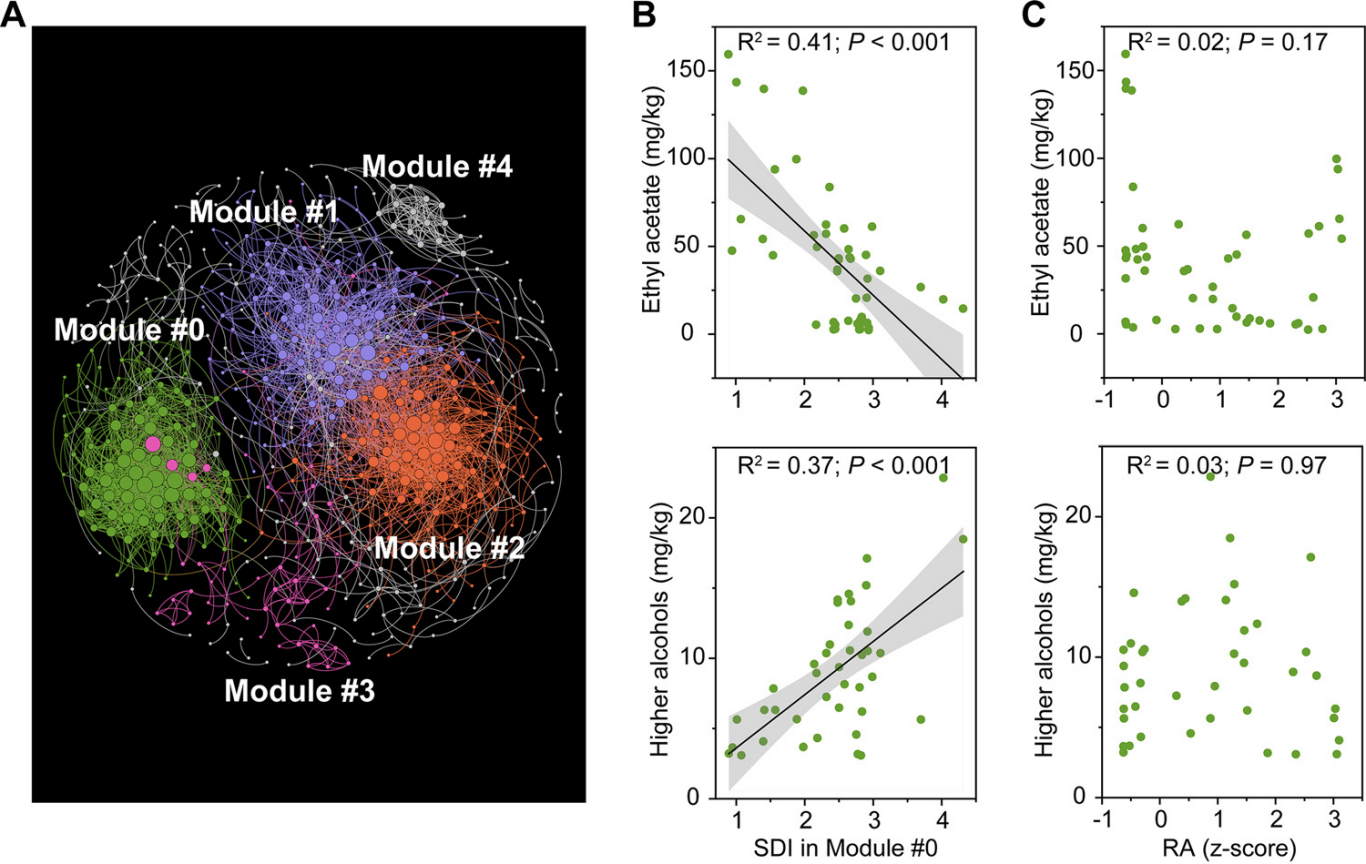
Keystone taxa linked to CSFB metabolites. (A) Network diagram with nodes colored according to each of the five main ecological clusters (modules 0 to 4). A total of 48 fermented grain samples were used to construct the network. (B) Regression relationships between the Shannon diversity index (SDI) of the microbial community in module 0 and the metabolite content. (C) Regression relationships between the relative abundance (RA) (z-score) of the microbial community in module 0 and the metabolite content.

The ecological network was clearly grouped into five main ecological clusters (modules 0 to 4) (Fig. 5A; see also Table S9 at https://doi.org/10.6084/m9.figshare.21382410). Over these modules, OTUs in module 0 had significantly higher degree and closeness centrality values than those of the other ecological clusters in the network (see Fig. S9A to C at https://doi.org/10.6084/m9.figshare.21379119), suggesting their importance in the microbial network. More importantly, we found that the SDI of the microbial community in module 0 was negatively and positively correlated with the levels of ethyl acetate and higher alcohols, respectively (Fig. 5B). In other words, we found a potential keystone microbial community belonging to module 0 that accounted for the most variations in the metabolites. More microbial ecological clusters that correlated with CSFB metabolite concentrations could be identified by considering more CSFB metabolites (Fig. S4). The concentrations of esters and acids were negatively correlated with the SDIs of the microbial communities only in module 0 and module 2, respectively (Fig. S4A and B). The alcohol concentration was negatively correlated with the module 0 SDI but positively correlated with the module 1 and module 2 SDIs (Fig. S4C). The aldehyde concentration was negatively correlated with module 1 and module 2 SDIs (Fig. S4D). Taken together, these results demonstrated the presence of potential keystone ecological clusters with specialized metabolic functions in CSFB fermentations.

Across all modules, only modules 0, 1, and 3 consisted of both bacteria and fungi (Fig. S5A). Module 0 showed a more diversified taxonomic distribution and was dominated by Thermoascus aurantiacus (OTU354), Weissella confusa (OTU34), and Aspergillus amstelodami (OTU2366 and OTU2610). In contrast, module 3 was dominated by only *Kazachstania africana* (OTU97) and *L. fermentum* (OTU6270, OTU7920, and OUT7590) (Fig. S6; see also Table S9 at the URL mentioned above). In particular, we found that keystone taxa and dominant taxa were clustered in module 0 and module 3, respectively. The relative abundances of bacteria and fungi were not significantly different in module 0 but varied significantly in module 3, implying that changes in the relative abundances of keystone taxa were not necessary for metabolite variations (Fig. S5B).

### Reconstruction of communities *in vitro* confirms the importance of keystone taxa in metabolic profile variation.

**FIG 6:**
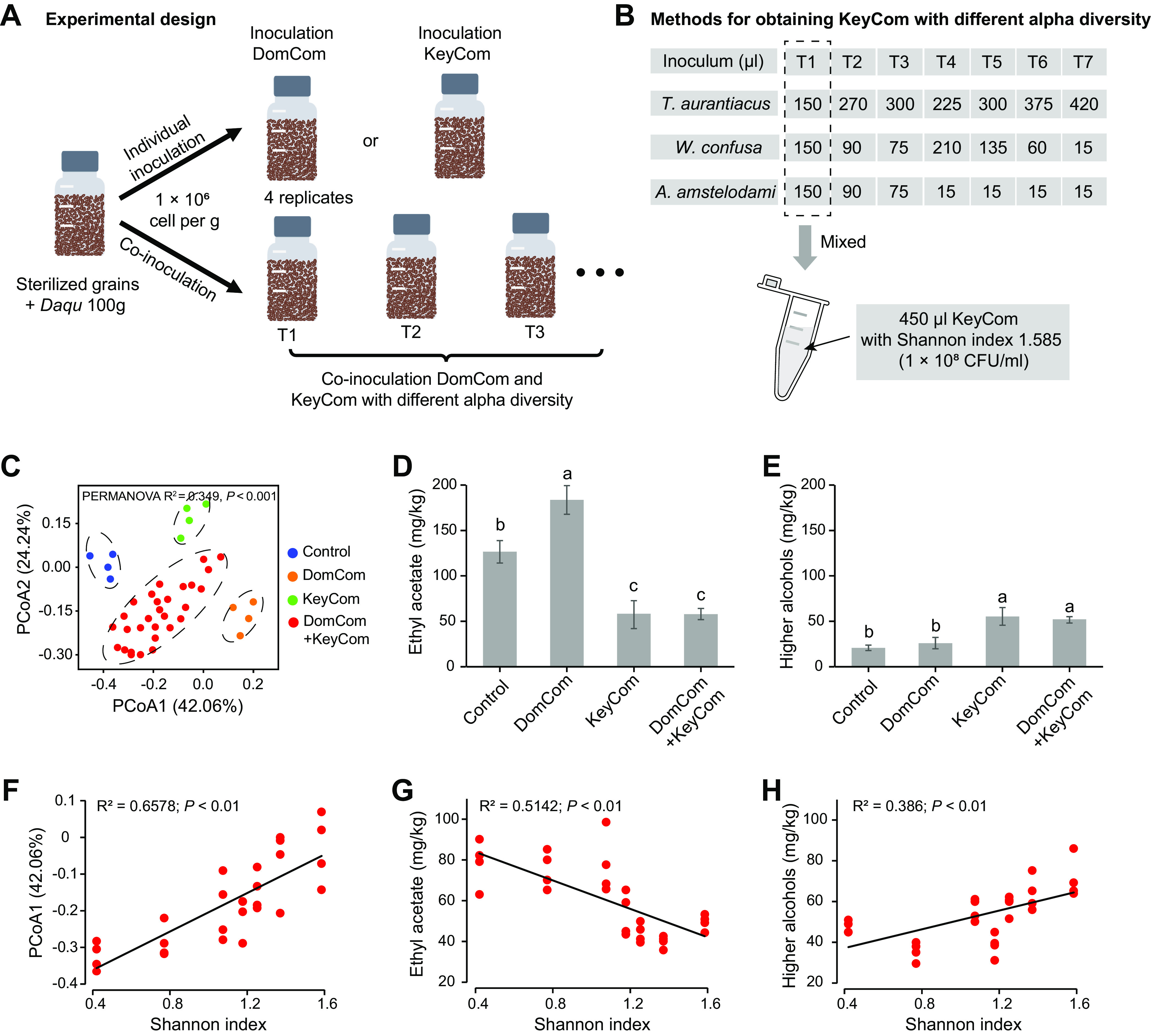
Validation of the important role of keystone taxa in metabolic profile variations. (A) Experimental setup for the *in vitro* system inoculated with DomCom or KeyCom alone or coinoculated with DomCom and KeyCom. (B) Methods for obtaining KeyCom with different alpha diversities. First, the three keystone species were cultured separately, and their cell densities were adjusted to about 1 × 10^8^ CFU/mL. We then mixed these keystone species according to the volumes in the table to obtain KeyCom with different alpha diversities. (C) PCoA visualizing metabolite variations in fermented grains (*n* = 40) after 30 days of fermentation. (D and E) Concentrations of ethyl acetate (D) and higher alcohols (E) in fermented grains. (F to H) Regression relationships between the Shannon index of KeyCom and metabolic profiles (represented by the PCoA1 axis) (F), the concentration of ethyl acetate (G), and the concentration of higher alcohols (H). Error bars in panels D and E represent the means ± standard deviations. Lowercase letters above the boxes indicate a significant difference at a *P* value of <0.05 by Tukey’s test.

In this study, we used a system-level approach to identify the dominant and keystone taxa responsible for metabolic variations during CSFB fermentation and found a strong correlation between the alpha diversities of keystone taxa and metabolite concentrations. To experimentally demonstrate the important role of keystone taxa in metabolic profile variations, we constructed a synthetic community containing dominant taxa (DomCom) or keystone taxa (KeyCom). DomCom consisted of four representative strains belonging to *L. fermentum*, *K. africana*, S. cerevisiae, and *P. kudriavzevii*. KeyCom consisted of three representative strains belonging to *Thermoascus aurantiacus*, *Weissella confusa*, and Aspergillus *amstelodami*. We inoculated them individually or in groups into the *in vitro* system (Fig. 6A) and then analyzed the resulting flavor profiles of the fermented grains after 30 days of fermentation. According to the PCoA results, there were considerable differences in the flavor profiles of fermented grains that had been individually inoculated with DomCom or KeyCom, coinoculated with DomCom and KeyCom, and uninoculated (*R*^2^ = 0.349; *P < *0.001 [by PERMANOVA]) (Fig. 6C). KeyCom decreased the concentration of ethyl acetate and increased the concentration of higher alcohols, while DomCom increased the ethyl acetate concentration (Fig. 6D and E). These results showed that both dominant taxa and keystone taxa affected the resulting flavor profiles.

To further evaluate the impact of the alpha diversity of keystone taxa on metabolic profile variations, we further constructed KeyCom with different alpha diversities (Fig. 6B). The resulting flavor profiles represented by the PCoA1 axis strongly correlated with the KeyCom Shannon index (Fig. 6F). In addition, the levels of ethyl acetate and higher alcohols were negatively and positively linked, respectively, with the KeyCom Shannon index (Fig. 6G and H), which was consistent with the results of the *in situ* sampling analysis. Combined, these results further confirm the importance of keystone taxa in metabolic profile variations.

### Ecological processes structuring the assemblies of the dominant and keystone taxa.

**FIG 7:**
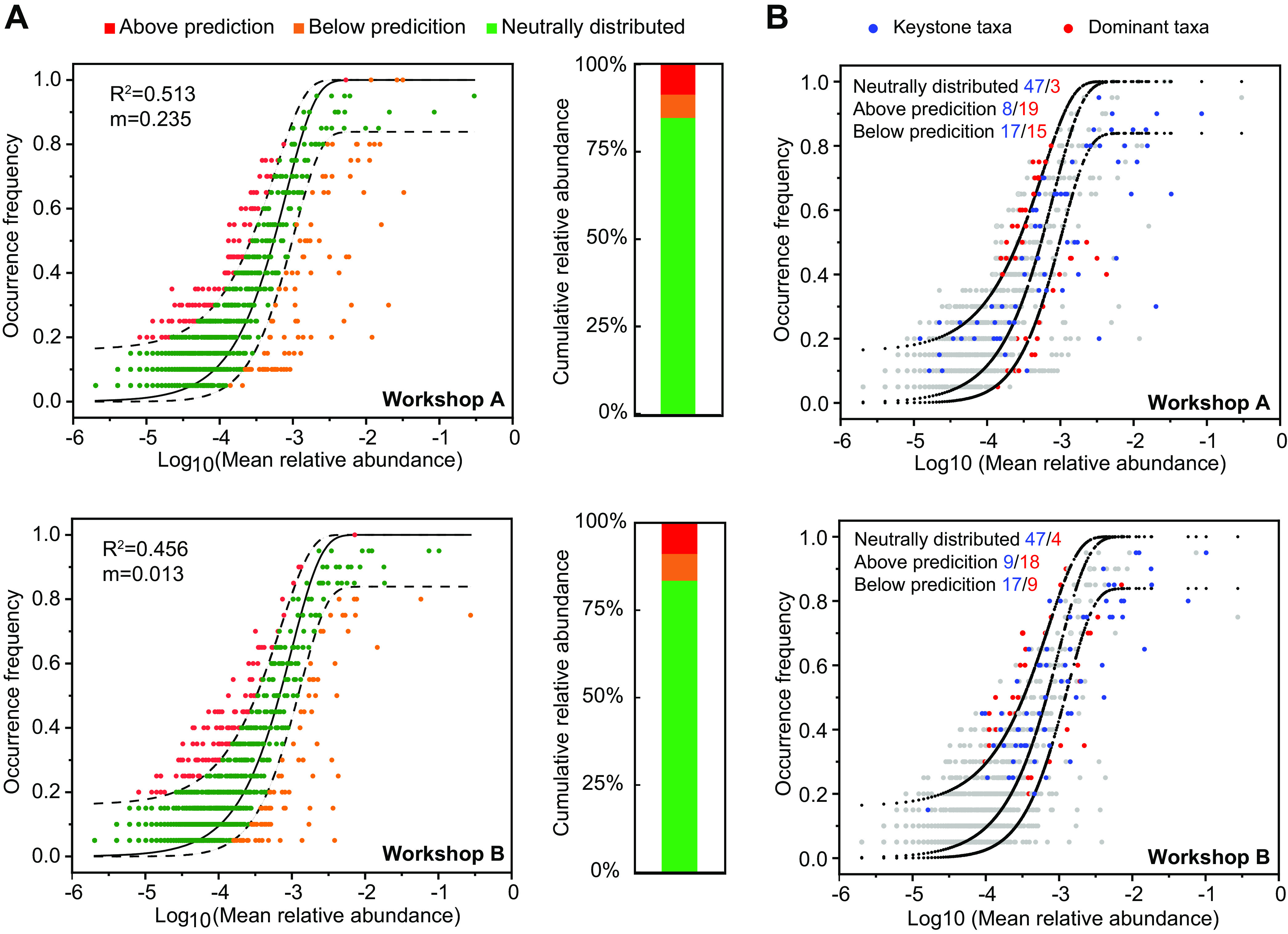
CSFB fermentation microbiota members assemble according to neutral rules. (A) Fit of the neutral models for microbial community assembly based on both bacteria and fungi from workshop A or B (*n* = 24 fermented grain samples for each workshop). The OTUs that occurred more frequently than predicted by the model are shown in red, while those that occurred less frequently than predicted are shown in orange. Black dashed lines represent the 95% confidence intervals around the model prediction, and the OTUs that fall within the confidence intervals are considered neutrally distributed. *R*^2^ values present the goodness of fit of the neutral model, ranging from 0 (no fit) to 1 (perfect fit). The histograms show the cumulative relative abundances of three categories of OTUs (above prediction, below prediction, and neutrally distributed). (B) Fit of the neutral models for the dominant and keystone taxa assemblies in workshop A or B. The dominant and keystone taxa are shown in red and blue, respectively. The numbers of keystone taxa or dominant taxa distributed into the three categories are shown in the top left corner of each graph.

To access the relative contribution of deterministic and stochastic processes to the CSFB microbiota assembly, we used the normalized stochasticity ratio (NST) index and the Sloan neutral model, which have previously been applied to the assemblies of many microbial communities (13, 15, 16, 42, 43). The NST_jac_ index (NST index based on the Jaccard distance) showed that microbes within both workshop A (NST_jac_ = 40.7%) and workshop B (NST_jac_ = 34.3%) were predominantly governed by deterministic processes. In addition, these observations suggested that deterministic processes increased while stochastic processes decreased in workshop B compared to workshop A (see Fig. S10A at https://doi.org/10.6084/m9.figshare.21379119). The NST based on Bray-Curtis dissimilarity (NST_bray_) also supported this result (38.4% and 32.4% for workshops A and B, respectively) (see Fig. S10B at https://doi.org/10.6084/m9.figshare.21379119). Although the deterministic process is dominant, the stochastic process also has a certain proportion that cannot be ignored. Next, we used the Sloan neutral model to describe the role of stochastic processes in microbial community construction. Overall, the Sloan neutral model estimated about half of the relationships between the occurrence frequencies of OTUs and their variations in relative abundance (Fig. 7A), with 51.3% and 45.6% of the community variance being explained for workshops A and B, respectively. The proportions of neutrally distributed taxa were 83.3% and 82.9% for workshops A and B, respectively. Additionally, the cumulative relative abundance of neutrally distributed OTUs was 80% in workshop A, the same as that in workshop B, indicating the good description of the microbial community assemblages in each workshop by neutral-distribution-based models. The estimated migration rate (*m*) was higher in workshop A (*m* = 0.235) than in workshop B (*m* = 0.013), suggesting relatively higher microorganism dispersal among fermented grains in workshop A and stronger dispersal limitation in workshop B.

In order to determine the differences in the assembly processes of the dominant taxa and keystone taxa, we mapped the keystone taxa and the dominant taxa determined in the neutral model (Fig. 7B). For keystone taxa, the distribution ratios of three categories of OTUs (64.4%, 12.3%, and 23.3% for neutral and above and below prediction, respectively) in the neutral model were almost the same between workshops A and B, indicating the importance of stochastic processes in shaping the assembly of keystone taxa. For the dominant taxa, almost all of them were distributed outside the 95% confidence interval in both workshops, indicating the importance of deterministic processes in shaping the assembly of dominant taxa.

## DISCUSSION

### Dominant taxa drive the variation in metabolic profiles by virtue of their relative abundance during CSFB fermentation.

In the present study, the metabolic profiles during fermentation in workshops A and B showed distinct successional trajectories. Moreover, EA was identified as an indicator metabolite for metabolic profile separation (Fig. 1). One explanation is that the heterogeneous environment during fermentation shapes distinct microbial community compositions, resulting in metabolic profile variations (19, 30). As expected, a Mantel test illustrated that temperature drove the microbial community composition and flavor profile variations between workshops A and B (see Fig. S11 at https://doi.org/10.6084/m9.figshare.21379119). *K. africana*, S. cerevisiae, and *P. kudriavzevii* were more abundant in workshop B (Fig. 2). Previous research has shown that these species can produce large amounts of ethyl acetate (44). This resulted in higher levels of ethyl acetate in workshop B. Another potential explanation for the metabolic profile changes is that the microbiota adjusted metabolic flux in response to environmental pressure. In the metabolic network, pyruvate acts as an intermediate hub. In principle, pyruvate can be converted to acetyl-CoA by pyruvate dehydrogenase and subsequently converted to EA by alcohol or ethanol *O*-acetyltransferases (36, 37); additionally, pyruvate can be converted to HAs through the anabolic pathway in cases of an insufficient amino-nitrogen supply (45, 46). In our study, the low-level expression of enzymes involved in the anabolic pathway was typically accompanied by the high-level expression of alcohol *O*-acetyltransferases (Fig. 3). This may be a microbiota metabolism strategy; that is, the microbiota regulates metabolic flux and produces the more volatile EA to reduce the pressure of ethanol or HAs during CSFB fermentation (47). However, intermediate metabolism should be assessed at the metabolome level in further studies to validate these findings. Similar to many previous studies that have assessed the important role of the core microbiome in driving ecosystem functions (48–50), we also identified homologous dominant taxa that carry genes with functions that were essential for metabolic profiles. These dominant taxa can affect microbiome function simply by their own high abundance (10). Collectively, our result indicated that the variation in metabolic profiles is directly related to the dominant taxa and can be affected exclusively by virtue of their relative abundances.

### Keystone taxa exert their influence on metabolic profiles irrespective of their abundance during CSFB fermentation.

The division of the network into modules can provide insights into the different groups of nodes that perform different functions (3, 42). Our microbial network was clearly classified into five ecological clusters (modules 0 to 4), and module 0 (dominated by *Thermoascus*, *Weissella*, and Aspergillus) was identified as a keystone ecological cluster (Fig. 5A). *Thermoascus*, *Weissella*, and Aspergillus are derived from Daqu (fermentation starter) or the environment (22), and they could secrete α-amylase and glucoamylase, which are essential for starch saccharification during pit fermentation (21). Furthermore, we found that the diversity of keystone taxa in module 0 largely determines CSFB flavor profiles (Fig. 5; see also Fig. S4 in the supplemental material). Moreover, the reconstruction of synthetic communities *in vitro* provided evidence that these keystone taxa and their diversity have the ability to modulate CSFB flavor profiles (Fig. 6). Recent studies have emphasized the important role of the diversity of keystone taxa in maintaining ecosystem multifunctionality (2, 8, 51) and high-level ecosystem function (e.g., organic matter decomposition and denitrification and crop yields) (3, 10). Those studies explained that the loss of the diversity of keystone taxa reduced functional redundancy (8) and the occurrences of specialized metabolic functional genes (11). In addition, many studies (11, 52) and our study found that the relative abundance of keystone taxa is generally low (<1%) (see Fig. S8 at https://doi.org/10.6084/m9.figshare.21379119), indicating the minor influence of their abundance. Therefore, the diversity of keystone taxa, rather than their relative abundance, is more likely to drive the CSFB community composition and flavor profiles.

EA and HAs were identified as indicator metabolites, and their concentrations showed opposite trends in response to changes in the diversity of keystone taxa (Fig. 5B). Previous studies have proposed that metabolic functions with different broadnesses (i.e., how many steps or diverse microbial groups are involved) respond differently to keystone taxa (10, 11). Keystone taxa might have a greater influence on narrow processes, that is, processes involving a single step and a small microbial group (10). Compared with broad processes (e.g., carbohydrate metabolism), the metabolic processes of indicator metabolites (EA and HAs) in our research are more likely to be narrow processes because only one or a few steps and simple microbiota were involved (Fig. 3). Therefore, the impact on these metabolites would be more pronounced when the keystone taxa were interfered with. In addition, keystone taxa, unlike other taxa, are expected to create a niche for other taxa and strongly support the structure of ecological networks, thus influencing ecosystem functions (48). Keystone taxa might first exert an influence to promote or suppress intermediate groups and then drive the community composition and metabolic functions (10). Such selective modulation is a ubiquitous regulation strategy (53). We speculate that the loss of keystone taxa in workshop B might stimulate the preemption of the ecological niche by EA-producing species (e.g., *K. africana* and *P. kudriavzevii*), while HA-producing species (e.g., S. cerevisiae) may be inhibited. Collectively, these findings highlight the importance of the diversity of keystone taxa within microbial communities in maintaining CSFB flavor profiles, providing the possibility of improving the quality of CSFB by regulating keystone taxa during fermentation.

### The CSFB microbial community assembly is shaped by stochastic and deterministic processes.

The normalized stochasticity ratio (NST) results suggested that stochastic and deterministic processes were responsible for shaping the microbial communities in CSFB fermentation simultaneously and that deterministic processes played a marginally larger role in controlling community assembly (see Fig. S10 at https://doi.org/10.6084/m9.figshare.21379119). Such a result is consistent with the findings of numerous previous studies claiming deterministic process-dominated community assembly but also emphasizing the positive role of stochastic processes. Previous studies suggested that niche-based selection can regulate the microbial community structure and flavor compound formation in CSFB fermentation caused by deterministic fitness differences between microorganisms (19). Such niche-based selection might include abiotic environmental selection (e.g., temperature, moisture, pH, anaerobic environment, and raw materials) (30, 54) and biotic interaction selection (e.g., facilitation between yeast and lactic acid bacteria [LAB]) (55). A Mantel test showed that temperature and moisture play important roles in shaping the microbial composition in workshop A but had a weak influence on that in workshop B (see Fig. S11A at https://doi.org/10.6084/m9.figshare.21379119). This result seems to be contrary to the NST results, which showed that the intensity of deterministic processes is stronger in workshop B than in workshop A. This may be attributed to unmeasured environmental variables, which include the dissolved oxygen concentration, the sampling location, and the operating environment (19, 22). For example, the environmental microbiota in different operating environments may colonize the fermented grain in the pit and subsequently affect microbial succession and metabolic profiles (22). Therefore, great caution is needed when using correlation or dimensionality reduction analyses such as the Mantel test, redundancy analysis (RDA), and variant partitioning analysis (VPA) to analyze the role of deterministic processes in community assembly, especially when the explained community variation is small (56). In this study, we applied NST analysis (43) to help quantitatively assess the relative importance of ecological deterministic processes. This method does not relate community composition to environmental variables and therefore can overcome the shortcomings of the Mantel test, RDA, and VPA.

### Differential community assembly processes of the dominant and keystone taxa.

Here, we applied the neutral community model to quantify the importance of processes that are difficult to observe directly but can have a large influence on microbial communities (i.e., dispersal and ecological drift). The Sloan neutral model explained about 50% of the detection frequency for bacteria and fungi from CSFB fermentation. Moreover, both the proportions and cumulative relative abundances of the neutral-distribution taxa exceeded 80% (Fig. 7A). These results provide evidence for the good fit of the neutral model for the CSFB microbiota. The neutral model separated taxa into neutral and nonneutral partitions. We found that the keystone taxa were distributed mainly in the prediction zone, whereas almost all of the dominant taxa showed a nonneutral distribution (Fig. 7B). This means that different ecological processes mediate the assemblies of keystone and dominant taxa. In this study, the assembly of keystone taxa showed a greater relative influence of stochastic processes (Fig. 7B). This may be due to the small population size of the keystone taxa because low-abundance microorganisms are more vulnerable to slight negative changes in their abundance (57). Another potential explanation is that the high level of diversity of keystone taxa induced functional redundancy, which may reduce fitness differences among cooccurring species (58), thereby weakening the role of niche selection.

### Conclusions.

In summary, we demonstrate that CSFB fermentations with distinct metabolic profiles display distinct microbial community compositions and microbial network complexities and stabilities. We then identified the dominant taxa (*L. fermentum*, *Kazachstania africana*, Saccharomyces cerevisiae, and *Pichia kudriavzevii*) and the keystone ecological cluster (module 0, affiliated mainly with *Thermoascus aurantiacus*, *Weissella confusa*, and Aspergillus *amstelodami*) that cause changes in metabolic profiles. We emphasized that both the relative abundance of the dominant taxa and the alpha diversity of the keystone taxa could contribute to the variations in the metabolic profiles. In addition, our results based on the NST index and the neutral model revealed that stochastic and deterministic processes together shaped the CSFB microbial community assembly. Stochasticity and environmental selection structure the keystone and dominant taxa, respectively. To the best of our knowledge, this study is the first to distinguish the metabolic functions and assembly processes of the keystone and dominant taxa in CSFB fermentation. Although our multi-omics analysis strategy focused only on metabolic function in CSFB fermentation, it should also be applicable to other ecological functions and food fermentation microecosystems. Our results are important because they open the door for isolating and applying these microbial communities to improve the controllability of food fermentation.

## MATERIALS AND METHODS

### Experimental design and sample collection.

The experimental site is located at Wuliangye Yibin Co., Ltd., in Sichuan Province, China. Six batches of fermentation with the same raw materials, operational conditions, and Daqu were selected for sample collection in workshops A and B in the winter of 2019. Based on the results of a previous study, the first 30 days are important for the fermentation process and flavor formation (23). Using a dense-to-sparse sampling strategy from the start to the middle of fermentation, 6 sampling time points (days 0, 2, 5, 10, 15, and 30) were set to collect fermented grains (see Fig. S1A in the supplemental material). Four parallel samples (100 g) were taken from two layers at each fermentation time point, and the same layers were mixed to make one sample (Fig. S1B). A total of 72 fermented grain samples were collected and immediately sent to the laboratory for subsequent physicochemical parameter determination, metabolite detection, and total DNA and RNA extraction (22).

### Environmental parameters and metabolite detection.

The temperature of fermented grains was measured *in situ* and recorded before sample collection. For moisture detection, about 10 g of fermented grain was dried at 105°C for 3 h, and weight loss was then measured. The total titratable acidity of the samples was determined by titration with a standard NaOH (0.1 mol/L) solution. Ethanol, acetate, and lactate were analyzed according to procedures described previously by Wang et al. (59). Their contents are shown in Tables S3 to S5 at https://doi.org/10.6084/m9.figshare.21382410. The flavor compound content was determined by headspace solid-phase microextraction gas chromatography-mass spectrometry (HS-SPME-GC-MS). Detailed methods reported previously by Wang et al. were used (59).

### Total DNA extraction, amplification, Illumina sequencing, and sequence processing.

Approximately 50-g samples were treated with sterile phosphate-buffered saline (PBS) and then centrifuged at 300 × *g* for 10 min (4°C). The supernatant was then centrifuged again at 13,000 × *g* for 10 min (4°C). The precipitated cells were milled with liquid nitrogen (50), and genomic DNA was extracted using sodium laurate buffer (sodium laurate at 10 g/L, Tris-HCl at 0.1 mol/L, NaCl at 0.1 mol/L, and EDTA at 0.02 mol/L) containing phenol-chloroform-isoamyl alcohol (25:24:1, vol/vol/vol) (50). The V3-V4 region of the bacterial 16S rRNA gene was amplified using the universal primers 338F (5′-ACTCCTACGGGAGGCAGCAG-3′) and 806R (5′-GACTACHVGGGTWTCTAAT-3′) (60). For fungi, the ITS2 region was amplified using the primers ITS3 (5′-GCATCGATGAAGAACGCAGC-3′) and ITS4 (5′-TCCTCCGCTTATTGATATGC-3′) (61). PCR products for each sample were purified using a PCR purification kit, and the concentrations were quantified using a Quant-It Pico green kit (Invitrogen, USA). The barcoded PCR products were sequenced on a MiSeq benchtop sequencer (Illumina, San Diego, CA) for 250-bp paired-end sequencing (2× 250 bp) at Beijing Auwigene Tech, Ltd. (Beijing, China).

All of the raw FASTQ files were processed using QIIME v1.9.1, and finally, the sequences were clustered into operational taxonomic units (OTUs) (97% sequence similarity cutoff). The representative bacterial and fungal OTU sequences (most abundant) were annotated using the EzBioCloud (version 20210707) and UNITE (version 9.0) databases with a QIIME-based wrapper of RDP Classifier (v2.2), respectively. The full data set after the above-described procedures contained 1,700,741 clean reads (mean of 41,481 reads per sample) for bacteria and 1,915,516 clean reads (mean of 43,534 reads per sample) for fungi.

### Total RNA extraction and metatranscriptome sequencing.

Total RNA was extracted from fermented grains according to procedures described previously by Song et al. (50). The RNA concentration was measured using a NanoDrop2000 instrument (NanoDrop Technologies, Wilmington, DE), and the RNA quality was measured using an Agilent 2200 instrument (Agilent Technologies, Santa Clara, CA, USA). Only samples with an RNA integrity number (RIN) value of ≥7 were used for subsequent library construction and sequencing (see Table S6 at https://doi.org/10.6084/m9.figshare.21382410). mRNA was isolated using magnetic beads, and oligo(dT) was then applied for metatranscriptome library construction using the NEBNext Ultra RNA library prep kit for Illumina (New England BioLabs). Sequencing of the metatranscriptome libraries was conducted at Beijing Auwigene Tech, Ltd. (Beijing, China), using a MiSeq benchtop sequencer (Illumina, San Diego, CA).

Quality control of the raw sequencing data is required, including the removal of low-quality reads, the removal of adapters, and calculations of the sequencing error rate, *Q*_20_ and *Q*_30_ statistics, and GC content, etc. An average of 32,609,177 raw reads were generated per sample. Each sample has an average of 23,037,899 clean reads and 3.46 G base amount (see Table S7 at https://doi.org/10.6084/m9.figshare.21382410). Trinity (v2.5.1) and CD-HIT (version 4.7) software were employed for *de novo* transcriptomic assemblies and spliced to obtain non-redundant gene sets (62). For functional annotation, these gene sets were aligned using BLASTX (version 2.5.0) with protein and nucleotide databases, including the Nonredundant Protein (NR), Pfam, Swiss-Prot, Kyoto Encyclopedia of Genes and Genomes (KEGG), Clusters of Orthologous Groups (COG), Gene Ontology (GO), and Carbohydrate-Active Enzymes (CAZy) databases (E value of <10^−5^), and identified according to the highest similarity to known sequences.

### Microbial network construction, characterization, and stability analyses.

Network analysis was performed to identify possible keystone taxa and assess microbial community complexity and stability. The construction of microbial networks was achieved in three steps. The first step was the selection of OTUs as nodes of the network. Only OTUs in >20% of all samples and only OTUs with an average relative abundance of >0.01% were selected. In total, 634 OTUs (546 bacterial OTUs and 88 fungal OTUs) were used. The second step was the selection of significant and strong relationships as edges. Only pairwise correlations with an absolute coefficient number (|ρ|) value of ≥0.8 and a *P* value of ≤0.01 were selected. The *P* values were adjusted by the Benjamini-Hochberg false discovery rate (FDR) test (63). The third step was the calculation and visualization of network properties. Network topology properties were calculated using the igraph R package (64). Modularity algorithms, including Fast greedy (65), Fast unfolding (66), and Near linear time (67), were used for module detection. Finally, the result with the highest modularity was selected and visualized using the Gephi interactive platform (WebAtlas, Paris, France).

Keystone taxa in networks were identified by their within-module connectivity (*Z_i_*) and among-module connectivity (*P_i_*). Based on criteria used in previous studies (41, 68), we identified network hubs (*Z_i_* of ≥2.5 and *P_i_* of ≥0.62), module hubs (*Z_i_* of ≥2.5 and *P_i_* of <0.62), connectors (*Z_i_* of <2.5 and *P_i_* of ≥0.62), and peripherals (*Z_i_* of <2.5 and *P_i_* of <0.62). Network hubs are nodes that are highly connected both within and between modules, module hubs are nodes that are highly connected within a module, and connectors are nodes that are highly connected between modules. All of these nodes can be referred to as keystone taxa (69, 70).

Network stability is quantified by robustness, which is defined as the proportion of the remaining species in this network after random or targeted node removal (41). According to the R codes reported previously by Yuan et al. (41), we made simple modifications and performed 200 simulations of random node removal with a ratio ranging from 0.5% to 1.

### *In vitro* construction of the synthetic community and solid-state fermentation experiments.

Based on the identification of dominant and keystone taxa in fermented grains, four dominant species (*L. fermentum*, *K. africana*, S. cerevisiae, and *P. kudriavzevii*) and three keystone species (T. aurantiacus, W. confusa, and A. amstelodami) that were isolated from fermented grains were used for the construction of DomCom and KeyCom, respectively. *L. fermentum*, *P. kudriavzevii*, S. cerevisiae, and *T. aurantiacus* were deposited in the China General Microbiological Culture Collection Center (CGMCC) with strain names 1.122, 12418, 2.3973, and 3.17992, respectively. *K. africana*, *W. confusa*, and *A. amstelodami* are laboratory strains WLY-F1, WLY-B1, and WLY-F2, respectively. *L. fermentum* and *W. confusa* were incubated in de Man-Rogosa-Sharpe (MRS) medium (Difco, Detroit, MI, USA) at 37°C for 24 h. *K. africana*, S. cerevisiae, *P. kudriavzevii*, and *A. amstelodami* were incubated in yeast extract-peptone-dextrose (YPD) medium at 30°C for 48 h. *T. aurantiacus* was incubated in potato dextrose broth (PDB) at 50°C for 48 h. Their cell densities were adjusted to about 1 × 10^8^ CFU/mL. Cell suspensions of *L. fermentum*, *K. africana*, S. cerevisiae, and *P. kudriavzevii* were mixed in equal volumes (150 mL) to obtain DomCom. *T. aurantiacus*, *W. confusa*, and *A. amstelodami* were mixed according to the volumes in Fig. 6B to obtain KeyCom with different alpha diversities.

Solid-state fermentation was performed under laboratory conditions to evaluate the effects of DomCom and KeyCom on the resulting flavor profiles. Solid medium was prepared according to methods described previously by Wang et al., using sorghum, corn, wheat, rice, and glutinous rice as raw materials (59). DomCom and KeyCom were individually inoculated or coinoculated into 250-mL bottles, which contained 100 g of sterilized grains and Daqu (Fig. 6A). The bottles were then sealed and incubated at 30°C. All fermentations were performed with 4 biological replicates. After 30 days of fermentation, the fermented grain samples were used to detect flavor compounds.

### Neutral model for microbial community assembly.

We applied the normalized stochasticity ratio (NST) to help quantitatively assess the relative importance of ecological deterministic and stochastic processes. NST was an index developed with 50% as the boundary point between more deterministic (<50%) and more stochastic (>50%) assemblies (43). The R package NST was used to calculate the index. The parameters were set as follows: dist.method of bray (or jaccard), abundance.weighted of TRUE, and rand of 1000. NST based on community similarity metrics, including the Jaccard distance (NST_jac_) and Bray-Curtis dissimilarity (NST_bray_), was calculated (data not shown). The Sloan neutral model (71) was used to determine the potential contribution of neutral processes in structuring the dominant and keystone taxa. This model predicts the relationship between the OTU detection frequency in a set of local communities (in this case, individual sample communities) and their relative abundance across the metacommunity (the communities of all samples). In general, the model predicts that the species with high abundances in the metacommunity are more likely to disperse by chance among different samples, while the species with low abundances are more likely to be lost due to ecological drift (72). The parameters *R*^2^ and *m* represent the overall fit to the neutral model and the immigration rate, respectively. The 95% confidence intervals around the fitting were calculated by bootstrapping with 1,000 bootstrap replicates. The OTUs were subsequently sorted into three partitions: (i) neutrally distributed, OTUs that occur within the 95% confidence interval, where the assembly of these OTUs is considered to be driven by stochastic processes; (ii) above prediction, OTUs that occur more frequently than the 95% confidence interval and are likely to have adapted to the fermentation environment or to have a stronger dispersal ability than others; and (iii) below prediction, OTUs that occur less frequently than the 95% confidence interval and are likely to be inhibited by the local environment or dispersal limited from the source community. All of the above-described operations were carried out in R using the R codes reported previously by Burns et al. (73).

### Statistical analysis.

Analysis of variance (ANOVA) and pairwise *t* tests were used to compare significant differences in physicochemical variables, the relative abundances of dominant microbial taxa, alpha diversity values, the abundances of functional genes, and the relative abundances of keystone taxa using SPSS statistical software (SPSS, Chicago, IL, USA). Alpha diversity indices were calculated in QIIME v1.8 using the alpha_diversity.py script. We used permutational multivariate analysis of variance (PERMANOVA) using distance matrices to evaluate the effects of a number of important variables on the metabolic profiles, including fermentation times, different workshops, and sampling locations. PERMANOVAs were carried out using the adonis function in the vegan package, using Bray-Curtis dissimilarities and 1,000 permutations. We identified indicator metabolites that are important for variations in metabolic profiles by the random-forest method. The importance of each metabolite was evaluated according to the mean decrease in accuracy. Tenfold cross-validation was performed using the rfcv function for selecting appropriate features (74). Mantel tests were used to infer the main environmental forces driving the differences in microbial community compositions. The relationships between the alpha diversity of keystone taxa and the concentration of metabolites were then tested by linear regressions. PCoA plots were generated using the ggplot2 package in R (version 3.2.0).

### Data availability.

The 16S rRNA gene sequence data have been deposited in the NCBI Sequence Read Archive (SRA) database under BioProject accession numbers PRJNA782866 and PRJNA783070. The metatranscriptome sequence data have been deposited in the NCBI SRA database under BioProject accession number PRJNA783532.

## References

[1] Patnode ML, Beller ZW, Han ND, Cheng J, Peters SL, Terrapon N, Henrissat B, Le Gall S, Saulnier L, Hayashi DK, Meynier A, Vinoy S, Giannone RJ, Hettich RL, Gordon JI. 2019. Interspecies competition impacts targeted manipulation of human gut bacteria by fiber-derived glycans. Cell 179:59–73.e13. doi:10.1016/j.cell.2019.08.011. 31539500 PMC6760872

[2] Saleem M, Hu J, Jousset A. 2019. More than the sum of its parts: microbiome biodiversity as a driver of plant growth and soil health. Annu Rev Ecol Evol Syst 50:145–168. doi:10.1146/annurev-ecolsys-110617-062605.

[3] Fan K, Delgado-Baquerizo M, Guo X, Wang D, Zhu Y-G, Chu H. 2021. Biodiversity of key-stone phylotypes determines crop production in a 4-decade fertilization experiment. ISME J 15:550–561. doi:10.1038/s41396-020-00796-8. 33028975 PMC8027226

[4] Toju H, Peay KG, Yamamichi M, Narisawa K, Hiruma K, Naito K, Fukuda S, Ushio M, Nakaoka S, Onoda Y, Yoshida K, Schlaeppi K, Bai Y, Sugiura R, Ichihashi Y, Minamisawa K, Kiers ET. 2018. Core microbiomes for sustainable agroecosystems. Nat Plants 4:247–257. doi:10.1038/s41477-018-0139-4. 29725101

[5] Kastman EK, Kamelamela N, Norville JW, Cosetta CM, Dutton RJ, Wolfe BE. 2016. Biotic interactions shape the ecological distributions of *Staphylococcus* species. mBio 7:e01157-16. doi:10.1128/mBio.01157-16. 27795388 PMC5082897

[6] Wolfe BE, Dutton RJ. 2015. Fermented foods as experimentally tractable microbial ecosystems. Cell 161:49–55. doi:10.1016/j.cell.2015.02.034. 25815984

[7] Fierer N. 2017. Embracing the unknown: disentangling the complexities of the soil microbiome. Nat Rev Microbiol 15:579–590. doi:10.1038/nrmicro.2017.87. 28824177

[8] Wagg C, Schlaeppi K, Banerjee S, Kuramae EE, van der Heijden MGA. 2019. Fungal-bacterial diversity and microbiome complexity predict ecosystem functioning. Nat Commun 10:4841. doi:10.1038/s41467-019-12798-y. 31649246 PMC6813331

[9] Hanson CA, Fuhrman JA, Horner-Devine MC, Martiny JB. 2012. Beyond biogeographic patterns: processes shaping the microbial landscape. Nat Rev Microbiol 10:497–506. doi:10.1038/nrmicro2795. 22580365

[10] Banerjee S, Schlaeppi K, van der Heijden MGA. 2018. Keystone taxa as drivers of microbiome structure and functioning. Nat Rev Microbiol 16:567–576. doi:10.1038/s41579-018-0024-1. 29789680

[11] Xun W, Liu Y, Li W, Ren Y, Xiong W, Xu Z, Zhang N, Miao Y, Shen Q, Zhang R. 2021. Specialized metabolic functions of keystone taxa sustain soil microbiome stability. Microbiome 9:35. doi:10.1186/s40168-020-00985-9. 33517892 PMC7849160

[12] Yue H, Zhang Y, He Y, Wei G, Shu D. 2019. Keystone taxa regulate microbial assemblage patterns and functional traits of different microbial aggregates in simultaneous anammox and denitrification (SAD) systems. Bioresour Technol 290:121778. doi:10.1016/j.biortech.2019.121778. 31310866

[13] Li P, Li W, Dumbrell AJ, Liu M, Li G, Wu M, Jiang C, Li Z. 2020. Spatial variation in soil fungal communities across paddy fields in subtropical China. mSystems 5:e00704-19. doi:10.1128/mSystems.00704-19. 31911465 PMC6946795

[14] Adair KL, Wilson M, Bost A, Douglas AE. 2018. Microbial community assembly in wild populations of the fruit fly Drosophila melanogaster. ISME J 12:959–972. doi:10.1038/s41396-017-0020-x. 29358735 PMC5864213

[15] Sprockett DD, Martin M, Costello EK, Burns AR, Holmes SP, Gurven MD, Relman DA. 2020. Microbiota assembly, structure, and dynamics among Tsimane horticulturalists of the Bolivian Amazon. Nat Commun 11:3772. doi:10.1038/s41467-020-17541-6. 32728114 PMC7391733

[16] Tong X, Leung MHY, Wilkins D, Cheung HHL, Lee PKH. 2019. Neutral processes drive seasonal assembly of the skin mycobiome. mSystems 4:e00004-19. doi:10.1128/mSystems.00004-19. 30944878 PMC6435813

[17] Zhou J, Ning D. 2017. Stochastic community assembly: does it matter in microbial ecology? Microbiol Mol Biol Rev 81:e0002-17. doi:10.1128/MMBR.00002-17. PMC5706748 29021219

[18] Jin G, Zhu Y, Xu Y. 2017. Mystery behind Chinese liquor fermentation. Trends Food Sci Technol 63:18–28. doi:10.1016/j.tifs.2017.02.016.

[19] Wu Q, Zhu Y, Fang C, Wijffels RH, Xu Y. 2021. Can we control microbiota in spontaneous food fermentation? Chinese liquor as a case example. Trends Food Sci Technol 110:321–331. doi:10.1016/j.tifs.2021.02.011.

[20] Hu X, Tian R, Wang K, Cao Z, Yan P, Li F, Li X, Li S, He P. 2021. The prokaryotic community, physicochemical properties and flavors dynamics and their correlations in fermented grains for Chinese strong-flavor Baijiu production. Food Res Int 148:110626. doi:10.1016/j.foodres.2021.110626. 34507770

[21] Zou W, Zhao C, Luo H. 2018. Diversity and function of microbial community in Chinese strong-flavor Baijiu ecosystem: a review. Front Microbiol 9:671. doi:10.3389/fmicb.2018.00671. 29686656 PMC5900010

[22] Wang X, Du H, Zhang Y, Xu Y. 2018. Environmental microbiota drives microbial succession and metabolic profiles during Chinese liquor fermentation. Appl Environ Microbiol 84:e02369-17. doi:10.1128/AEM.02369-17. 29196296 PMC5795089

[23] Wang X, Du H, Xu Y. 2017. Source tracking of prokaryotic communities in fermented grain of Chinese strong-flavor liquor. Int J Food Microbiol 244:27–35. doi:10.1016/j.ijfoodmicro.2016.12.018. 28064120

[24] Liu H, Sun B. 2018. Effect of fermentation processing on the flavor of Baijiu. J Agric Food Chem 66:5425–5432. doi:10.1021/acs.jafc.8b00692. 29751730

[25] Wang S, Xiong W, Wang Y, Nie Y, Wu Q, Xu Y, Geisen S. 2020. Temperature-induced annual variation in microbial community changes and resulting metabolome shifts in a controlled fermentation system. mSystems 5:e00555-20. doi:10.1128/mSystems.00555-20. 32694129 PMC7566281

[26] Fu J, Chen L, Yang S, Li Y, Jin L, He X, He L, Ao X, Liu S, Liu A, Yang Y, Ma B, Cui X, Chen S, Zou L. 2021. Metagenome and analysis of metabolic potential of the microbial community in pit mud used for Chinese strong-flavor liquor production. Food Res Int 143:110294. doi:10.1016/j.foodres.2021.110294. 33992393

[27] Hu X, Wang K, Chen M, Fan J, Han S, Hou J, Chi L, Liu Y, Wei T. 2020. Profiling the composition and metabolic activities of microbial community in fermented grain for the Chinese strong-flavor Baijiu production by using the metatranscriptome, high-throughput 16S rRNA and ITS gene sequencings. Food Res Int 138:109765. doi:10.1016/j.foodres.2020.109765. 33292946

[28] Wang H, Gu Y, Zhou W, Zhao D, Qiao Z, Zheng J, Gao J, Chen X, Ren C, Xu Y. 2021. Adaptability of a caproate-producing bacterium contributes to its dominance in an anaerobic fermentation system. Appl Environ Microbiol 87:e01203-21. doi:10.1128/AEM.01203-21. 34378978 PMC8478455

[29] Wang X, Wang B, Sun Z, Tan W, Zheng J, Zhu W. 2022. Effects of modernized fermentation on the microbial community succession and ethyl lactate metabolism in Chinese Baijiu fermentation. Food Res Int 159:111566. doi:10.1016/j.foodres.2022.111566. 35940782

[30] Xiao C, Lu Z-M, Zhang X-J, Wang S-T, Ao L, Shen C-H, Shi J-S, Xu Z-H. 2017. Bio-heat is a key environmental driver shaping the microbial community of medium-temperature Daqu. Appl Environ Microbiol 83:e01550-17. doi:10.1128/AEM.01550-17. 28970223 PMC5691423

[31] Wang Z, Li P, Luo L, Simpson DJ, Ganzle MG. 2018. Daqu fermentation selects for heat-resistant Enterobacteriaceae and Bacilli. Appl Environ Microbiol 84:e01483-18. doi:10.1128/AEM.01483-18. 30120119 PMC6193390

[32] Wu Q, Kong Y, Xu Y. 2016. Flavor profile of Chinese liquor is altered by interactions of intrinsic and extrinsic microbes. Appl Environ Microbiol 82:422–430. doi:10.1128/AEM.02518-15. 26475111 PMC4711146

[33] Ma S, Luo H, Zhao D, Qiao Z, Zheng J, An M, Huang D. 2022. Environmental factors and interactions among microorganisms drive microbial community succession during fermentation of Nongxiangxing Daqu. Bioresour Technol 345:126549. doi:10.1016/j.biortech.2021.126549. 34902488

[34] Pang X-N, Han B-Z, Huang X-N, Zhang X, Hou L-F, Cao M, Gao L-J, Hu G-H, Chen J-Y. 2018. Effect of the environment microbiota on the flavour of light-flavour Baijiu during spontaneous fermentation. Sci Rep 8:3396. doi:10.1038/s41598-018-21814-y. 29467508 PMC5821866

[35] Zhang H, Du H, Xu Y. 2021. Volatile organic compound-mediated antifungal activity of *Pichia* spp. and its effect on the metabolic profiles of fermentation communities. Appl Environ Microbiol 87:e02992-20. doi:10.1128/AEM.02992-20. 33608301 PMC8091005

[36] Kruis AJ, Levisson M, Mars AE, van der Ploeg M, Garces Daza F, Ellena V, Kengen SWM, van der Oost J, Weusthuis RA. 2017. Ethyl acetate production by the elusive alcohol acetyltransferase from yeast. Metab Eng 41:92–101. doi:10.1016/j.ymben.2017.03.004. 28356220

[37] Holt S, Trindade de Carvalho B, Foulquie-Moreno MR, Thevelein JM. 2018. Polygenic analysis in absence of major effector ATF1 unveils novel components in yeast flavor ester biosynthesis. mBio 9:e01279-18. doi:10.1128/mBio.01279-18. 30154260 PMC6113618

[38] Arskold E, Lohmeier-Vogel E, Cao R, Roos S, Radstrom P, van Niel EW. 2008. Phosphoketolase pathway dominates in *Lactobacillus reuteri* ATCC 55730 containing dual pathways for glycolysis. J Bacteriol 190:206–212. doi:10.1128/JB.01227-07. 17965151 PMC2223725

[39] Pires EJ, Teixeira JA, Branyik T, Vicente AA. 2014. Yeast: the soul of beer’s aroma—a review of flavour-active esters and higher alcohols produced by the brewing yeast. Appl Microbiol Biotechnol 98:1937–1949. doi:10.1007/s00253-013-5470-0. 24384752

[40] Hammer SK, Avalos JL. 2017. Uncovering the role of branched-chain amino acid transaminases in *Saccharomyces cerevisiae* isobutanol biosynthesis. Metab Eng 44:302–312. doi:10.1016/j.ymben.2017.10.001. 29037781

[41] Yuan MM, Guo X, Wu L, Zhang Y, Xiao N, Ning D, Shi Z, Zhou X, Wu L, Yang Y, Tiedje JM, Zhou J. 2021. Climate warming enhances microbial network complexity and stability. Nat Clim Chang 11:343–348. doi:10.1038/s41558-021-00989-9.

[42] Du S, Dini-Andreote F, Zhang N, Liang C, Yao Z, Zhang H, Zhang D. 2020. Divergent co-occurrence patterns and assembly processes structure the abundant and rare bacterial communities in a salt marsh ecosystem. Appl Environ Microbiol 86:e00322-20. doi:10.1128/AEM.00322-20. 32358000 PMC7301849

[43] Ning D, Deng Y, Tiedje JM, Zhou J. 2019. A general framework for quantitatively assessing ecological stochasticity. Proc Natl Acad Sci USA 116:16892–16898. doi:10.1073/pnas.1904623116. 31391302 PMC6708315

[44] Zhang S, Guo F, Yan W, Dong W, Zhou J, Zhang W, Xin F, Jiang M. 2020. Perspectives for the microbial production of ethyl acetate. Appl Microbiol Biotechnol 104:7239–7245. doi:10.1007/s00253-020-10756-z. 32656615

[45] Jiang J, Liu Y, Li H, Yang Q, Wu Q, Chen S, Tang J, Xu Y. 2019. Modeling and regulation of higher alcohol production through the combined effects of the C/N ratio and microbial interaction. J Agric Food Chem 67:10694–10701. doi:10.1021/acs.jafc.9b04545. 31476866

[46] Hu Y, Yang Q, Chen D, Fu B, Zhang Y, Zhang Y, Xia X, Peng N, Liang Y, Zhao S. 2021. Study on microbial communities and higher alcohol formations in the fermentation of Chinese Xiaoqu Baijiu produced by traditional and new mechanical technologies. Food Res Int 140:109876. doi:10.1016/j.foodres.2020.109876. 33648194

[47] Loser C, Urit T, Bley T. 2014. Perspectives for the biotechnological production of ethyl acetate by yeasts. Appl Microbiol Biotechnol 98:5397–5415. doi:10.1007/s00253-014-5765-9. 24788328

[48] Stopnisek N, Shade A. 2021. Persistent microbiome members in the common bean rhizosphere: an integrated analysis of space, time, and plant genotype. ISME J 15:2708–2722. doi:10.1038/s41396-021-00955-5. 33772106 PMC8397763

[49] Shade A, Stopnisek N. 2019. Abundance-occupancy distributions to prioritize plant core microbiome membership. Curr Opin Microbiol 49:50–58. doi:10.1016/j.mib.2019.09.008. 31715441

[50] Song Z, Du H, Zhang Y, Xu Y. 2017. Unraveling core functional microbiota in traditional solid-state fermentation by high-throughput amplicons and metatranscriptomics sequencing. Front Microbiol 8:1294. doi:10.3389/fmicb.2017.01294. 28769888 PMC5509801

[51] Xun W, Li W, Xiong W, Ren Y, Liu Y, Miao Y, Xu Z, Zhang N, Shen Q, Zhang R. 2019. Diversity-triggered deterministic bacterial assembly constrains community functions. Nat Commun 10:3833. doi:10.1038/s41467-019-11787-5. 31444343 PMC6707308

[52] Banerjee S, Walder F, Büchi L, Meyer M, Held AY, Gattinger A, Keller T, Charles R, van der Heijden MGA. 2019. Agricultural intensification reduces microbial network complexity and the abundance of keystone taxa in roots. ISME J 13:1722–1736. doi:10.1038/s41396-019-0383-2. 30850707 PMC6591126

[53] Kommineni S, Bretl DJ, Lam V, Chakraborty R, Hayward M, Simpson P, Cao Y, Bousounis P, Kristich CJ, Salzman NH. 2015. Bacteriocin production augments niche competition by enterococci in the mammalian gastrointestinal tract. Nature 526:719–722. doi:10.1038/nature15524. 26479034 PMC4978352

[54] Tan Y, Zhong H, Zhao D, Du H, Xu Y. 2019. Succession rate of microbial community causes flavor difference in strong-aroma Baijiu making process. Int J Food Microbiol 311:108350. doi:10.1016/j.ijfoodmicro.2019.108350. 31614280

[55] Liu J, Wu Q, Wang P, Lin J, Huang L, Xu Y. 2017. Synergistic effect in core microbiota associated with sulfur metabolism in spontaneous Chinese liquor fermentation. Appl Environ Microbiol 83:e01475-17. doi:10.1128/AEM.01475-17. 28970229 PMC5717201

[56] Chen W, Ren K, Isabwe A, Chen H, Liu M, Yang J. 2019. Stochastic processes shape microeukaryotic community assembly in a subtropical river across wet and dry seasons. Microbiome 7:138. doi:10.1186/s40168-019-0749-8. 31640783 PMC6806580

[57] Nemergut DR, Schmidt SK, Fukami T, O’Neill SP, Bilinski TM, Stanish LF, Knelman JE, Darcy JL, Lynch RC, Wickey P, Ferrenberg S. 2013. Patterns and processes of microbial community assembly. Microbiol Mol Biol Rev 77:342–356. doi:10.1128/MMBR.00051-12. 24006468 PMC3811611

[58] Spasojevic MJ, Catano CP, Lamanna JA, Myers JA. 2018. Integrating species traits into species pools. Ecology 99:1265–1276. doi:10.1002/ecy.2220. 29569239

[59] Wang S, Wu Q, Nie Y, Wu J, Xu Y. 2019. Construction of synthetic microbiota for reproducible flavor compound metabolism in Chinese light-aroma-type liquor produced by solid-state fermentation. Appl Environ Microbiol 85:e03090-18. doi:10.1128/AEM.03090-18. 30850432 PMC6498162

[60] Soergel DA, Dey N, Knight R, Brenner SE. 2012. Selection of primers for optimal taxonomic classification of environmental 16S rRNA gene sequences. ISME J 6:1440–1444. doi:10.1038/ismej.2011.208. 22237546 PMC3379642

[61] Toju H, Tanabe AS, Yamamoto S, Sato H. 2012. High-coverage ITS primers for the DNA-based identification of ascomycetes and basidiomycetes in environmental samples. PLoS One 7:e40863. doi:10.1371/journal.pone.0040863. 22808280 PMC3395698

[62] Grabherr MG, Haas BJ, Yassour M, Levin JZ, Thompson DA, Amit I, Adiconis X, Fan L, Raychowdhury R, Zeng Q, Chen Z, Mauceli E, Hacohen N, Gnirke A, Rhind N, di Palma F, Birren BW, Nusbaum C, Lindblad-Toh K, Friedman N, Regev A. 2011. Trinity: reconstructing a full-length transcriptome assembly without a reference genome from RNA-Seq data. Nat Biotechnol 29:644–652. doi:10.1038/nbt.1883. 21572440 PMC3571712

[63] Benjamini Y, Hochberg Y. 1995. Controlling the false discovery rate: a practical and powerful approach to multiple testing. J R Stat Soc B 57:289–300. doi:10.1111/j.2517-6161.1995.tb02031.x.

[64] Csardi G. 2006. The igraph software package for complex network research. InterJ Complex Syst 1695:1–9.

[65] Clauset A, Newman MEJ, Moore C. 2004. Finding community structure in very large networks. Phys Rev E Stat Nonlin Soft Matter Phys 70:e066111. doi:10.1103/PhysRevE.70.066111. 15697438

[66] Blondel VD, Guillaume J-L, Lambiotte R, Lefebvre E. 2008. Fast unfolding of communities in large networks. J Stat Mech 2008:P10008. doi:10.1088/1742-5468/2008/10/P10008.

[67] Raghavan UN, Albert R, Kumara S. 2007. Near linear time algorithm to detect community structures in large-scale networks. Phys Rev E Stat Nonlin Soft Matter Phys 76:e036106. doi:10.1103/PhysRevE.76.036106. 17930305

[68] Shi Y, Delgado-Baquerizo M, Li Y, Yang Y, Zhu YG, Penuelas J, Chu H. 2020. Abundance of kinless hubs within soil microbial networks are associated with high functional potential in agricultural ecosystems. Environ Int 142:105869. doi:10.1016/j.envint.2020.105869. 32593837

[69] Rottjers L, Faust K. 2019. Can we predict keystones? Nat Rev Microbiol 17:193. doi:10.1038/s41579-018-0132-y. 30542201

[70] Banerjee S, Schlaeppi K, van der Heijden MGA. 2019. Reply to ‘Can we predict microbial keystones?’. Nat Rev Microbiol 17:194. doi:10.1038/s41579-018-0133-x. 30538306

[71] Sloan WT, Lunn M, Woodcock S, Head IM, Nee S, Curtis TP. 2006. Quantifying the roles of immigration and chance in shaping prokaryote community structure. Environ Microbiol 8:732–740. doi:10.1111/j.1462-2920.2005.00956.x. 16584484

[72] Wang Y, Wang K, Huang L, Dong P, Wang S, Chen H, Lu Z, Hou D, Zhang D. 2020. Fine-scale succession patterns and assembly mechanisms of bacterial community of *Litopenaeus vannamei* larvae across the developmental cycle. Microbiome 8:106. doi:10.1186/s40168-020-00879-w. 32620132 PMC7334860

[73] Burns AR, Stephens WZ, Stagaman K, Wong S, Rawls JF, Guillemin K, Bohannan BJ. 2016. Contribution of neutral processes to the assembly of gut microbial communities in the zebrafish over host development. ISME J 10:655–664. doi:10.1038/ismej.2015.142. 26296066 PMC4817674

[74] Jiang G, Wang W. 2017. Error estimation based on variance analysis of k-fold cross-validation. Pattern Recognit 69:94–106. doi:10.1016/j.patcog.2017.03.025.

