# Supplementary material for: Stochastic Processes Drive the Assembly and Metabolite Profiles of Keystone Taxa during Chinese Strong-Flavor Baijiu Fermentation
Source: Microbiol Spectr. 2023 Mar 14;11(2):e05103-22. doi: 10.1128/spectrum.05103-22 (PMC10101002; doi:10.1128/spectrum.05103-22)
Supplement: Supplemental file 1 — Table S1 and Fig. S1 to S6. Download spectrum.05103-22-s0001.pdf, PDF file, 1.1 MB [file spectrum.05103-22-s0001.pdf]

Supplementary materials

Stochastic Processes Drive Keystone Taxa Assembly and Metabolite Profiles during Chinese Strong-flavor Baijiu Fermentation

Shukun Yuan[a], Xiaowei Yu[a], Hai Du[a*], Dong Zhao[b], Zongwei Qiao[b], Jia Zheng[b], Yan Xu[a*]

[a]Laboratory of Brewing Microbiology and Applied Enzymology, Key Laboratory of Industrial Biotechnology of Ministry of Education, School of Biotechnology, Jiangnan University, 1800 Lihu Avenue, Wuxi, Jiangsu 214122, China

[b]Wuliangye Yibin Co., Ltd, 150# Minjiang West Road, Cuiping District, Yibin, Sichuan 644007, China

*** Corresponding author:**

Tel.: +86-510-85964112; Fax: +86-510-85918201.

E-mail address: yxu@jiangnan.edu.cn (Yan Xu); duhai88@126.com

This supplementary file includes:

Supplementary table: Table S1

Supplementary figures: Fig. S1-Fig. S6

Supplementary figures caption:

Fig. S1 Schema of the experimental design with collected data types and sample sizes. (A) Fermented grain samples (n=72) were collected from six batches of fermentation in workshop A and B at day 0, 2, 5, 10, 15 and 30. Points of different shapes represent different data types. Samples from the same workshop at the same time point (days 2, 5, 10, 15, 30) are mixed into one sample for metatranscriptome sequencing. (B) Sampling positions during the pit fermentation. The prefix “A” and “B” indicated that samples were collected from the upper and middle layers in pit fermentation respectively. One final sample was made by mixing samples from different points in the same layer for reducing the heterogeneity of samples.

Fig. S2 Microbial succession during fermentation from different workshops. Average bacterial (A) and fungal (C) distribution at the genus-level of microbiota. PCoA visualizing compositional variations of bacterial (B) and fungal (D) communities over fermentation time based on Bray-Curtis distance.

Fig. S3 Determination of the dominant microbiota. Taxonomic contributions to ethyl acetate (A, B), higher alcohols biosynthesis (C, D, E) and acetate (F) during CSFB fermentation. (G) The dominant species in the fermented grains based on amplicon (blue circle) and metatranscriptome sequencing (yellow circle). (H) The concentrations of ethyl acetate and higher alcohols produced by dominant species after 3 days fermentation in sorghum extract medium.

Fig. S4 The regression relationships between the microbial diversity of modules and the concentration of CSFB metabolites, including esters (A), acids (B), alcohols (C), and aldehydes (D).

Fig. S5 The richness proportion (A) and relative abundance (z-score, B) of bacteria and fungi at operational taxonomic unit (OTU) level in each ecological cluster. Different letters indicate a significant difference determined by non-parametric Kruskal–Wallis test.

Fig. S6 The dominant (average relative abundance > 0.5% in Module #0, #1, #2; average relative abundance > 0.1% in Module #3, #4) bacteria distribution (A), and dominant (average relative abundance > 0.1% in Module #0, #1, #3) fungi distribution (B) at genus level in each ecological cluster of the co-occurrence network.

Supplementary table

Table S1 Topological properties of the networks in workshop A and B

Network metric	Workshop A	Workshop B
Number of nodes	381	265
Number of links	1126	383
Clustering coefficient	0.566	0.433
Density	0.016	0.011
Modularity	0.821	0.793
No. of modules	80	69
No. of large modules [a]	13	9
No. of nodes in large modules [a]	295	136
Percentage of nodes in large modules (%) [a]	77.4	51.3
No. of nodes in the largest module	42	34
Fungal nodes (%)	18.11	13.58
Negative links (%)	2.49	2.11

[a] Large modules contain at least 5 nodes.

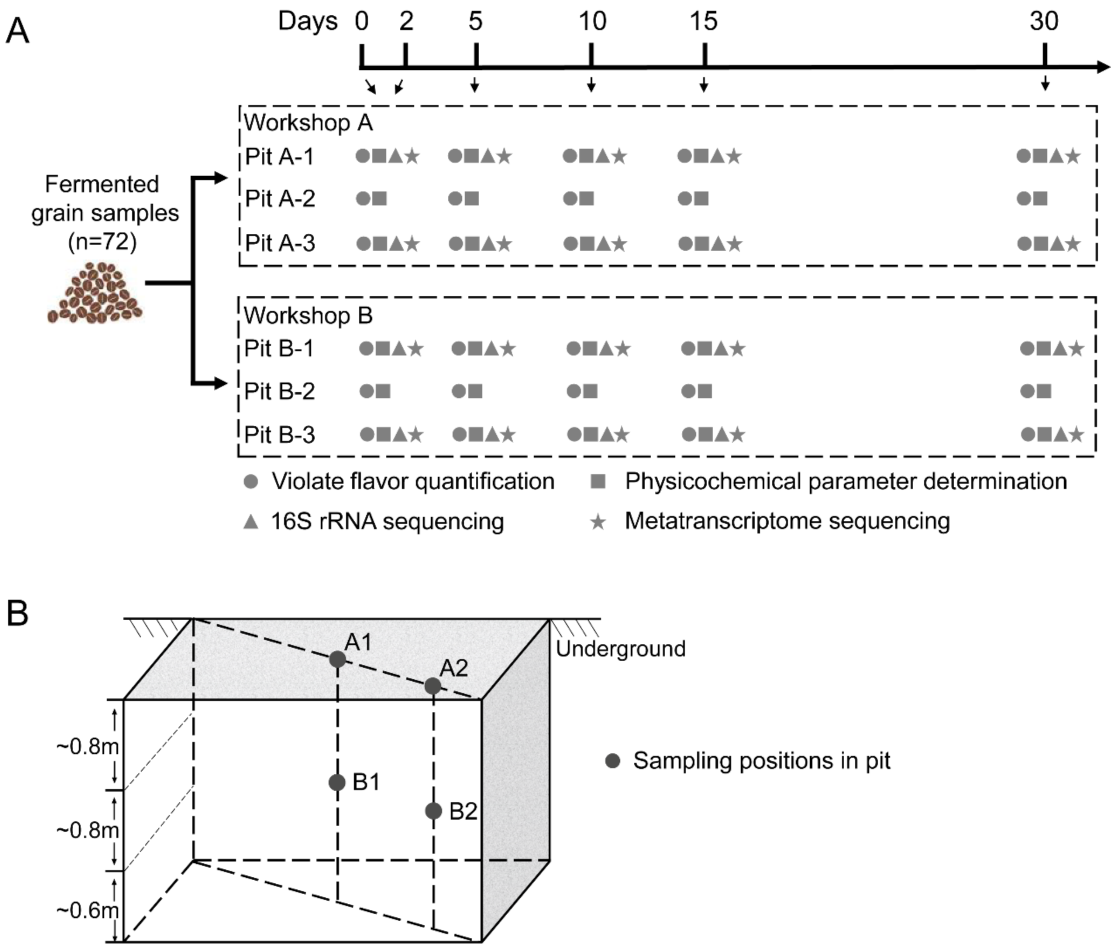
A
Days 0 2 5 10 15 30
Fermented grain samples (n=72)
Workshop A
Pit A-1
Pit A-2
Pit A-3
Workshop B
Pit B-1
Pit B-2
Pit B-3
Violate flavor quantification
Physicochemical parameter determination
16S rRNA sequencing
Metatranscriptome sequencing
B
A1
A2
Underground
B1
B2
~0.8m
~0.8m
~0.6m
Sampling positions in pit

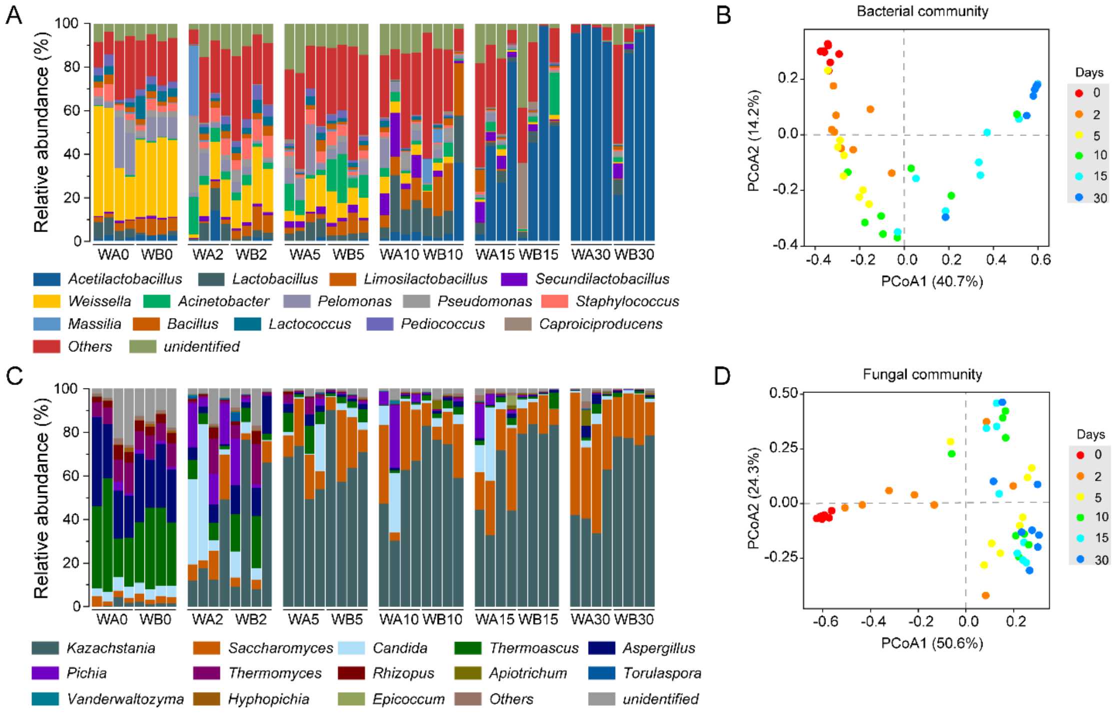
A
Relative abundance (%)
100
80
60
40
20
0
WA0 WB0
WA2 WB2
WA5 WB5
WA10 WB10
WA15 WB15
WA30 WB30
Acetilactobacillus
Lactobacillus
Limosilactobacillus
Secundilactobacillus
Weissella
Acinetobacter
Pelomonas
Pseudomonas
Staphylococcus
Massilia
Bacillus
Lactococcus
Pediococcus
Caproiciproducens
Others
unidentified
B
Bacterial community
PCoA2 (14.2%)
PCoA1 (40.7%)
0.2
0.0
-0.2
-0.4
-0.4
-0.2
0.0
0.2
0.4
0.6
Days
0
2
5
10
15
30
C
Relative abundance (%)
100
80
60
40
20
0
WA0 WB0
WA2 WB2
WA5 WB5
WA10 WB10
WA15 WB15
WA30 WB30
Kazachstania
Saccharomyces
Candida
Thermoascus
Aspergillus
Pichia
Thermomyces
Rhizopus
Apiotrichum
Torulaspora
Vanderwaltozyma
Hyphopichia
Epicoccum
Others
unidentified
D
Fungal community
PCoA2 (24.3%)
PCoA1 (50.6%)
0.50
0.25
0.00
-0.25
-0.6
-0.4
-0.2
0.0
0.2
Days
0
2
5
10
15
30

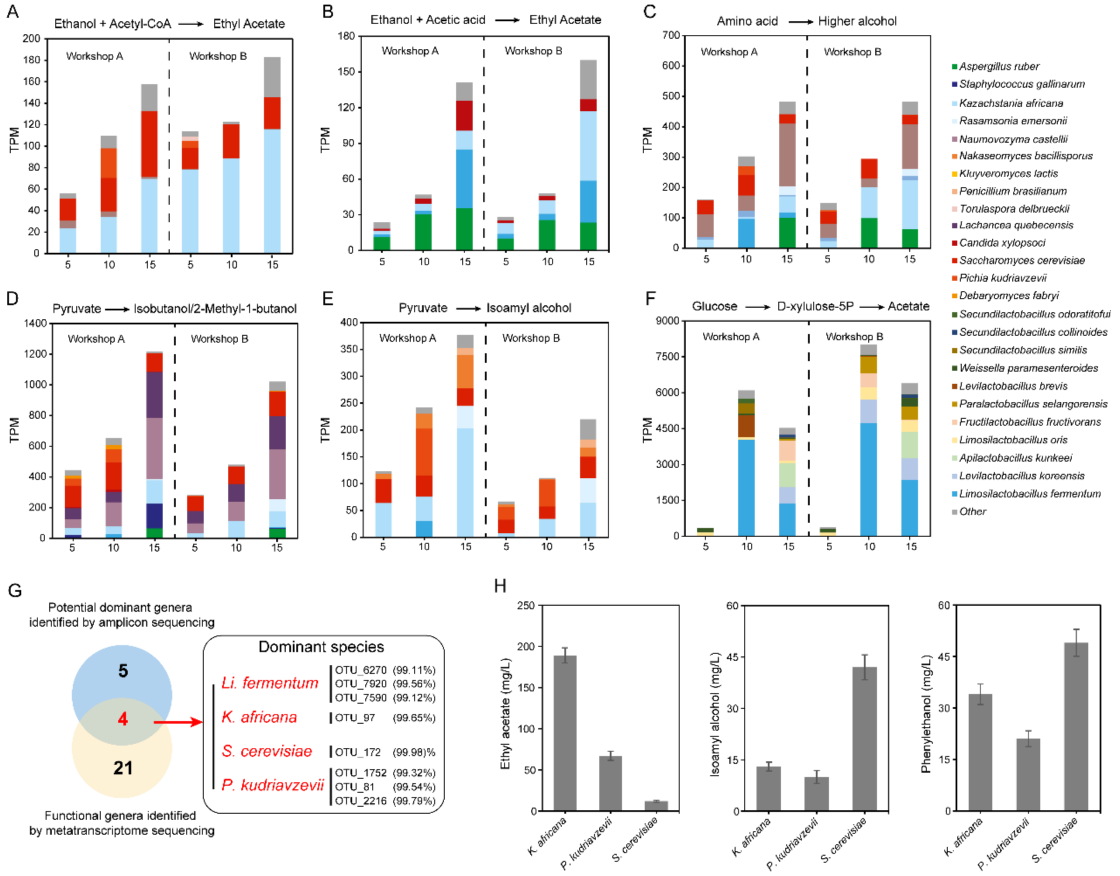
A
Ethanol + Acetyl-CoA → Ethyl Acetate
B
Ethanol + Acetic acid → Ethyl Acetate
C
Amino acid → Higher alcohol
D
Pyruvate → Isobutanol/2-Methyl-1-butanol
E
Pyruvate → Isoamyl alcohol
F
Glucose → D-xylulose-5P → Acetate
Workshop A
Workshop B
TPM
Aspergillus ruber
Staphylococcus gallinarum
Kazachstania africana
Rasamsonia emersonii
Naumovozyma castellii
Nakaseomyces bacillisporus
Kluyveromyces lactis
Penicillium brasilianum
Torulaspora delbrueckii
Lachancea quebecensis
Candida xylopsoci
Saccharomyces cerevisiae
Pichia kudriavzevii
Debaryomyces fabryi
Secundilactobacillus odoratitofui
Secundilactobacillus collinoides
Secundilactobacillus similis
Weissella paramesenteroides
Levilactobacillus brevis
Paralactobacillus selangorensis
Fructilactobacillus fructivorans
Limosilactobacillus oris
Apilactobacillus kunkeei
Levilactobacillus koreensis
Limosilactobacillus fermentum
Other
G
Potential dominant genera identified by amplicon sequencing
5
4
21
Functional genera identified by metatranscriptome sequencing
Dominant species
Li. fermentum
OTU_6270 (99.11%)
OTU_7920 (99.56%)
OTU_7590 (99.12%)
K. africana
OTU_97 (99.65%)
S. cerevisiae
OTU_172 (99.98)%
P. kudriavzevii
OTU_1752 (99.32%)
OTU_81 (99.54%)
OTU_2216 (99.79%)
H
Ethyl acetate (mg/L)
Isoamyl alcohol (mg/L)
Phenylethanol (mg/L)
K. africana
P. kudriavzevii
S. cerevisiae

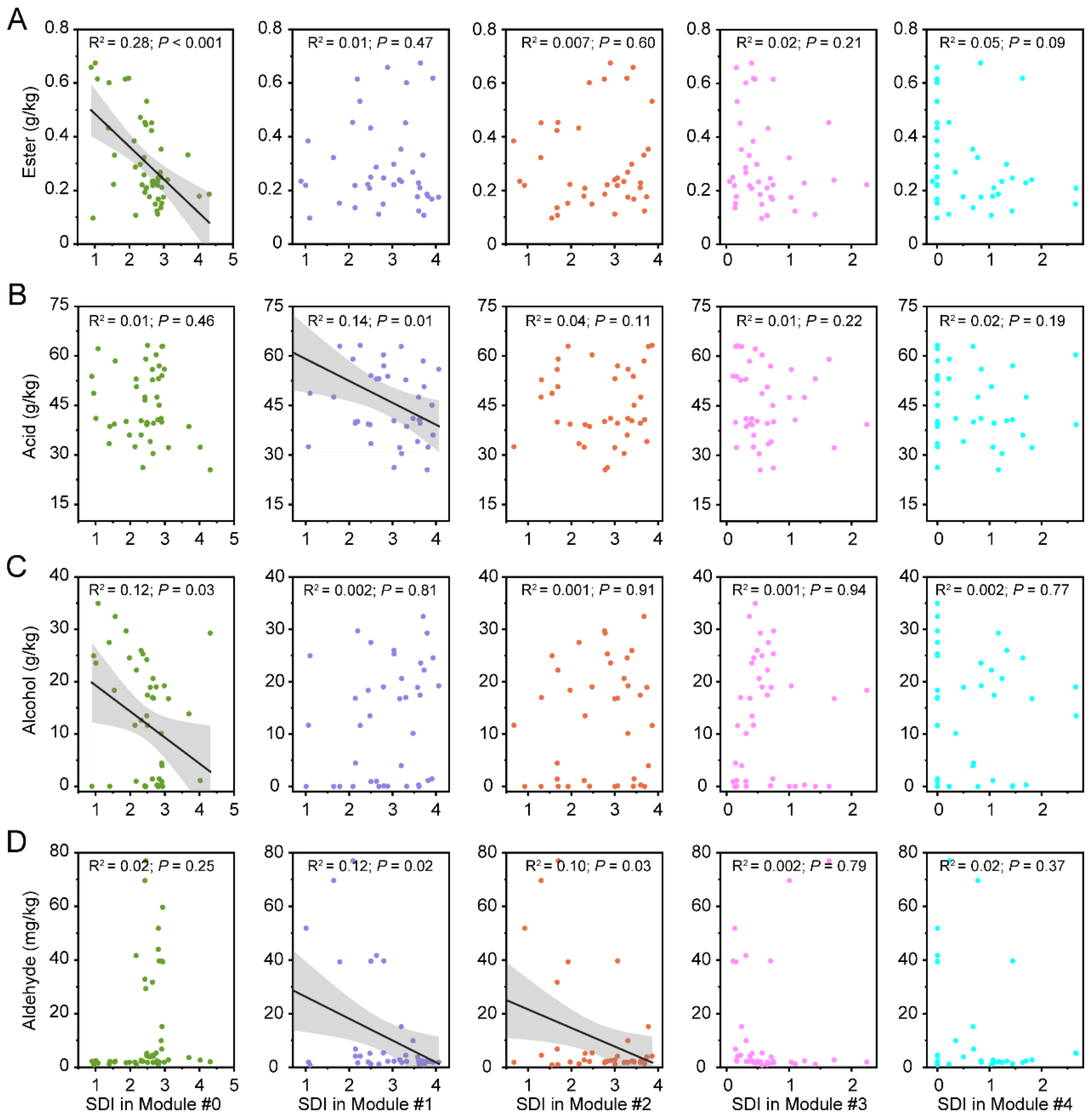

A
B
C
D
Ester (g/kg)
Acid (g/kg)
Alcohol (g/kg)
Aldehyde (mg/kg)
R² = 0.28; P < 0.001
R² = 0.01; P = 0.47
R² = 0.007; P = 0.60
R² = 0.02; P = 0.21
R² = 0.05; P = 0.09
R² = 0.01; P = 0.46
R² = 0.14; P = 0.01
R² = 0.04; P = 0.11
R² = 0.01; P = 0.22
R² = 0.02; P = 0.19
R² = 0.12; P = 0.03
R² = 0.002; P = 0.81
R² = 0.001; P = 0.91
R² = 0.001; P = 0.94
R² = 0.002; P = 0.77
R² = 0.02; P = 0.25
R² = 0.12; P = 0.02
R² = 0.10; P = 0.03
R² = 0.002; P = 0.79
R² = 0.02; P = 0.37
SDI in Module #0
SDI in Module #1
SDI in Module #2
SDI in Module #3
SDI in Module #4

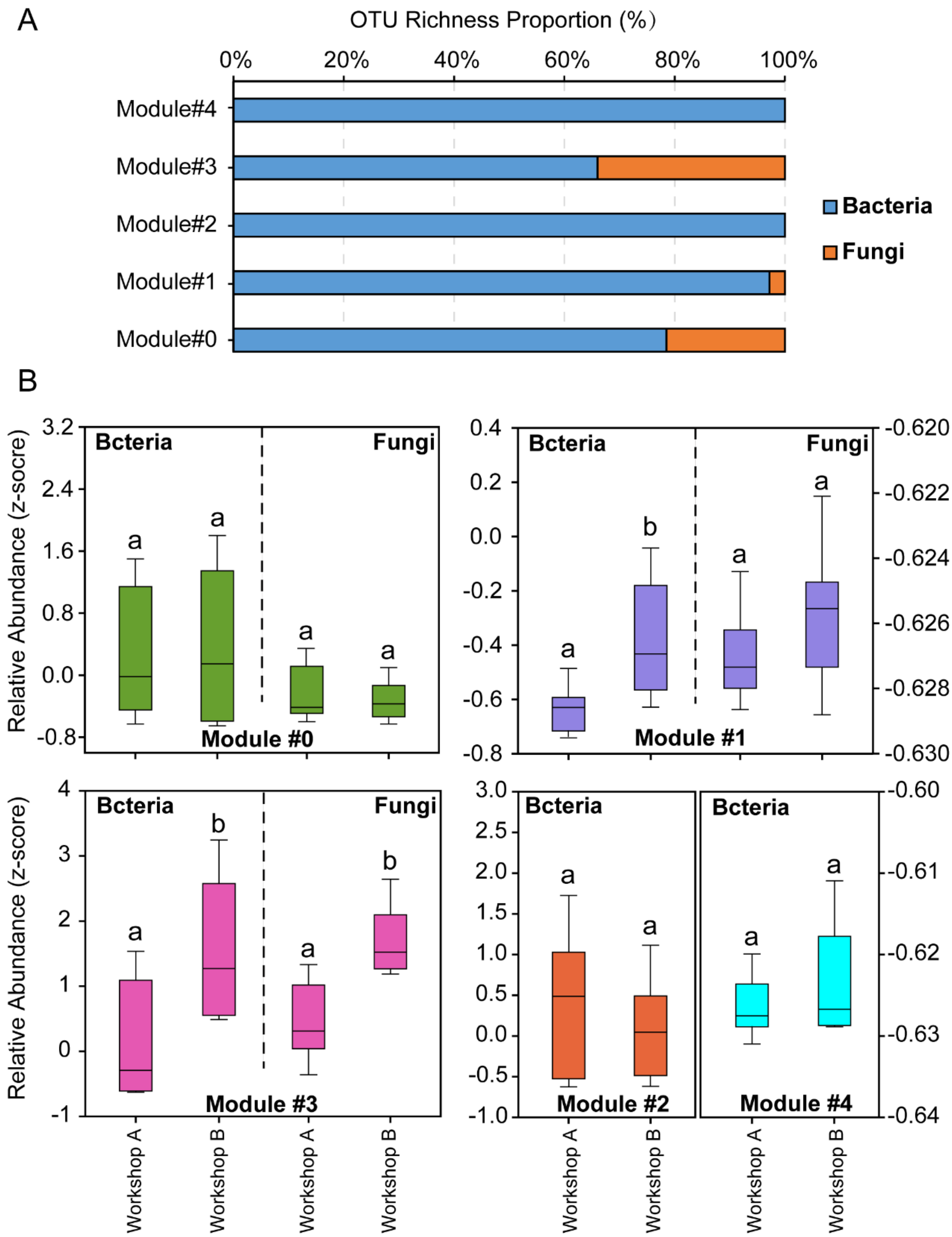
A
OTU Richness Proportion (%)
0%
20%
40%
60%
80%
100%
Module#4
Module#3
Module#2
Module#1
Module#0
Bacteria
Fungi
B
Relative Abundance (z-socre)
Bcteria
Fungi
Module #0
Module #1
Relative Abundance (z-score)
Module #3
Module #2
Module #4
Workshop A
Workshop B

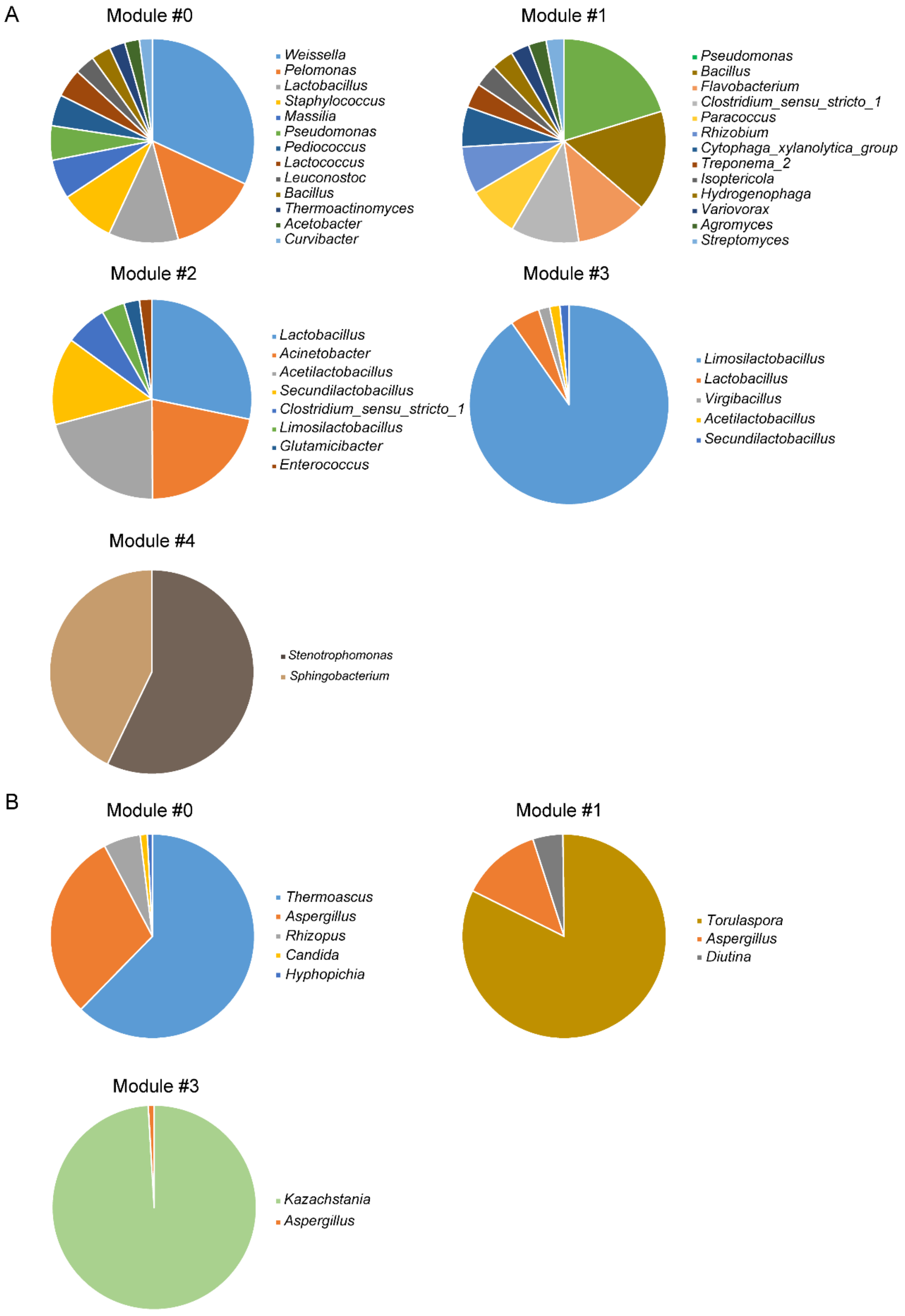
A
Module #0
Weissella
Pelomonas
Lactobacillus
Staphylococcus
Massilia
Pseudomonas
Pediococcus
Lactococcus
Leuconostoc
Bacillus
Thermoactinomyces
Acetobacter
Curvibacter
Module #1
Pseudomonas
Bacillus
Flavobacterium
Clostridium_sensu_stricto_1
Paracoccus
Rhizobium
Cytophaga_xylanolytica_group
Treponema_2
Isoptericola
Hydrogenophaga
Variovorax
Agromyces
Streptomyces
Module #2
Lactobacillus
Acinetobacter
Acetilactobacillus
Secundilactobacillus
Clostridium_sensu_stricto_1
Limosilactobacillus
Glutamicibacter
Enterococcus
Module #3
Limosilactobacillus
Lactobacillus
Virgibacillus
Acetilactobacillus
Secundilactobacillus
Module #4
Stenotrophomonas
Sphingobacterium
B
Module #0
Thermoascus
Aspergillus
Rhizopus
Candida
Hyphopichia
Module #1
Torulaspora
Aspergillus
Diutina
Module #3
Kazachstania
Aspergillus
